# Network Pharmacology Prediction and Molecular Docking-Based Strategy to Discover the Potential Pharmacological Mechanism of Wen-Yu-Jin against Pulmonary Fibrosis in a Mouse Model

**DOI:** 10.1155/2022/7753508

**Published:** 2022-02-10

**Authors:** Lu Wang, Wenxiang Zhu, Rui Sun, Jing Liu, Qihong Ma, Binbin Zhang, Yuanyuan Shi

**Affiliations:** ^1^School of Life Sciences, Beijing University of Chinese Medicine, Beijing, China; ^2^Shenzhen Research Institute, Beijing University of Chinese Medicine, Shenzhen, China

## Abstract

**Background:**

Pulmonary fibrosis (PF) is a devastating lung disease, resulting in gas exchange dysfunction until death. The two drugs approved by the FDA, pirfenidone and nintedanib, have obvious side effects. Wen-yu-jin (WYJ), one of the commonly used herbs in China, can treat respiratory diseases. The potential effects and the underlying mechanism of WYJ against PF are unclear.

**Purpose:**

Employing network pharmacology, molecular docking, and in vivo and in vitro experiments to explore the potential effects and underlying mechanisms of WYJ in the treatment of PF.

**Methods:**

Ultra-high pressure liquid chromatography combined with linear ion trap-orbital tandem mass spectrometry (UHPLC-LTQ-orbital trap) was used to identify compounds of WYJ. We got PF-related targets and WYJ compounds-related targets from public databases and further completed critical targets exploration, network construction, and pathway analysis by network pharmacology. Molecular docking predicted binding activity of WYJ compounds and critical targets. Based on the above results, in vivo and in vitro experiments validated the potential effects and mechanisms of WYJ against PF.

**Results:**

23 major compositions of WYJ were identified based on UHPLC-LTQ-Orbitrap. According to the results of network pharmacology, STAT3, SRC, IL6, MAPK1, AKT1, EGFR, MAPK8, MAPK14, and IL1B are critical therapeutic targets. Molecular docking results showed that most of the compounds have good binding activities with critical targets. The results of in vivo and in vitro experiments showed that WYJ alleviated the process of fibrosis by targeting MAPK and STAT3 pathways.

**Conclusion:**

Network pharmacology, molecular docking, and in vivo and in vitro experiments showed the potential effects and mechanisms of WYJ against PF, which provides a theoretical basis for the treatment of WYJ with PF.

## 1. Introduction

Pulmonary fibrosis (PF) characterized by progressive dyspnea with respiratory failure [1–3] is an irreversible lung disease [4]. PF susceptibility is closely related to age [5]. Accumulating exposures to numerous risk factors [6], such as smoking, occupational dust, drug stimulation, and bacterial and virus infection, also result in PF. Different risk factors repeatedly promote lung injury, leading to the production of profibrotic cytokines. TGF-*β*1 is one of the most potent profibrosis cytokines [7, 8]. Profibrotic cytokines further stimulate effector cells activation and migration, leading to the deposition of extracellular matrix (ECM). ECM, a typical feature of pulmonary fibrosis, affects the gas exchange function of the lung. They eventually lead to respiratory failure and death [9, 10]. Pirfenidone and nintedanib [11], approved by FDA, are the only two effective drugs for clinical PF medical therapies. However, these drugs can only delay the disease's progression and maintain lung function but cannot cure the disease [12, 13]. Moreover, these drugs can cause adverse effects [11, 13–14], resulting in treatment discontinuation and adverse gastrointestinal effects.

Multiple active ingredients and targets of Traditional Chinese Medicines (TCM) arouse the attention of pharmacologists. Previous studies showed that TCM or active ingredients from TCM have protective pulmonic [15], representing an attractive source of drug discovery for treating PF. In TCM, there are many theories about the pathogenesis of PF, such as qi stagnation and blood stasis (Qi-Zhi-Xue-Yu in Chinese) and binding of phlegm and qi (Tan-Qi-Hu-Jie in Chinese). Wen-yu-jin (WYJ, Curcuma Radix, 温郁金), derived from the steamed root of Curcuma beauty, has been used for at least 1500 years in China and has the effect of moving qi and blood circulation. Modern pharmacological studies have demonstrated that the ingredients or extracts from WYJ have antibacterial, antitumor anti-inflammatory, and antioxidant effects [16, 17]. Hui's study showed potential antifibrotic effects of WYJ in liver fibrosis [18]. Therefore, it is necessary to study the therapeutic effects and mechanism of WYJ in the treatment of PF using advanced methods.

In silico approaches have been widely used in the field of drug molecule design, improving efficiency and reducing cost [19, 20]. Based on bioinformatics, systems biology, and pharmacology, network pharmacology is a promising tool for understanding the complex relationship between drugs and diseases [21]. This tool has been successfully used to reveal the molecular mechanism of TCM in various diseases such as cardiovascular disease [22, 23], diabetes [24], and cancer [25]. Some approaches, such as molecular docking and molecular dynamics simulation (MDS), help researchers understand the pathogenesis of diseases at the molecular level and provide new ideas and theoretical guidance for the design of new drugs [20, 26]. In a study, researchers organized MDS to understand the resistance mechanism of mutant BCR-ABL protein, which provided ideas for the design of new drugs [27]. Rachel through in silico operations elucidated that Ligand2 has stronger inhibitory potential for cyclin-dependent kinases (CDKs) and can develop into a new selective inhibitor of CDK2 [19].

In our study, we established network pharmacology to explore the molecular mechanism of WYJ against PF. Molecular docking was performed to predict the affinity strength between the active compounds and the critical targets and to explore the antipulmonary fibrosis potential of WYJ. Finally, the antifibrosis mechanism of WYJ was further verified via in vitro and in vivo experiments based on the predicted results.

## 2. Materials and Methods

### 2.1. Preparation of Chinese Medicine

WYJ was purchased from Tong Ren Tang Co., Ltd. (Beijing, China). The herb was boiled in purified water twice for 1 h each time. We got lyophilized powder in condensation and freeze-dry of liquids and stored it at −80°C for a series of experiments.

### 2.2. LC/MS Analysis

We used processed WYJ for in vitro and in vivo experiments. The compounds of processed WYJ were reduced or changed compared with the herbal plant's library. Therefore, we used LC/MS analysis to clarify the compounds of WYJ. The coupling of the UHPLC System (Thermo Fisher Scientific) equipped with a binary pump, an auto sampler, a column thermostat, and DAD detector, and LTQ-Orbitrap XL (Thermo Fisher Scientific) equipped with an electrostatic ionization source (ESI) was used For LC/MS experiments. Data was controlled and processed by Xcalibur software (Thermo Fisher Scientific). The column used in the LC analysis was Thermo Scientific Hypersil BDS C18 (2.1 mm 150 mm, 2.4 *μ*m). Mobile phase A was water with 0.1% formic acid while mobile phase B was acetonitrile. The column temperature was set to 35 and the flow rate was 0.3 mL/min. Elution conditions were summarized as follows: 0–3 min (5%-5%B), 3–45 min (5%∼75%B, 45–45.1 min (75%-5%B), and 45.1–50 min (5%-5%B).

The MS analysis was conducted on both the negative and positive ion modes. Flow rates of sheath gas and auxiliary gas were set at 40 Arb and 20 Arb, respectively. The capillary voltage was set to 35.0 V. The source temperature was set to 350 and the tube lens was set to 110 V. The source voltage was set to 4 kV and 3 kV in the positive and negative ion modes, respectively.

### 2.3. Network Pharmacology

According to the compounds identified by UHPLC-LTQ-Orbitrap, we obtained these compound-related targets from the SwissTargetPrediction database [28] (http://www.swisstargetprediction.ch/) and Traditional Chinese Medicine Systems Pharmacology Database and Analysis Platform (TCMSP, https://tcmsp-e.com/). Compounds-related protein names were converted to the gene names using UniProt (https://www.uniprot.org/).

We used “pulmonary fibrosis (PF),” “idiopathic pulmonary fibrosis (IPF),” or “interstitial lung disease (ILD)” as keywords to obtain PF-related targets from GeneCards database v5.0 [29] (https://www.genecards) and OMIM database [30] (https://omim.org/). After combining the targets obtained from different databases using the same keywords, we got the targets of PF by taking the intersection of targets with different keywords.

WYJ-related targets and PF-related targets were intersected to obtain gene symbols. Using the string database (https://www.string-db.org/) [31], the protein-protein interaction (PPI) network was constructed to explore the potential interactions of the intersected gene symbols. Cytoscape v3.6.0 visualized the PPI network. Critical nodes calculated by the median values of three topological features (degree, closeness centrality, and bitterness centrality) were identified as key targets of WYJ.

To determine the biological meaning behind key targets, pathway enrichment was analyzed by Gene Ontology (GO) and the Kyoto Encyclopedia of Genes and Genomes (KEGG) database of David v6.8 (https://david.ncifcrf.gov/) [32, 33]. The threshold value of confident gene enrichment was set at *P* < 0.05. Compound-target-pathway network explored the connection of compounds of WYJ, key targets, and important signaling pathways and was visualized using Cytoscape.

### 2.4. Molecular Docking

The active compounds were molecularly docked with the core proteins based on the results of network pharmacology. The specific operations are as follows: (1) ligands molecule preparation. We downloaded the core compound structures from the Pubchem database (https://pubchem.ncbi.nlm.nih.gov/). All compounds were optimized using the MM2 force field, and saved in.pdbqt format as docking ligands in AutoDock Tools 1.5.6 software (ADT) [34]. (2) Receptor molecule preparation. The crystal structures of proteins were obtained from the RCSB PDB website (http://www.rcsb.org/). Proteins should be human proteins with one or more cocrystallized ligands and crystal structures with small “resolution” value. PyMoL software was utilized to remove water molecules and original ligands [35]. AutoDockTools software was used to add nonpolar hydrogen, calculate the Gasteiger charge, and assign the AD4 type. Receptors were ready and saved in.pdbqt format. (3) Molecular docking. A suitable method of evaluating the reliability of a docking procedure was needed. Prior to docking the ligands against the target proteins, the redocking of the cocrystal structures and their original ligands was carried out. All of the root mean square deviations (RMSDs) were less than 2 Å which indicated the method for docking was reasonable. The grid center of molecular docking was determined by using the cocrystallized ligand in the target protein complex. AutoDock Vina 1.1.2 was utilized for the docking of prepared ligands and proteins. And the spacing and exhaustiveness were set to 0.375 and 8, respectively. In particular, for IL-6 blind docking was performed with exhaustiveness 24. A Lamarckian genetic algorithm (LGA) was used for the conformational search. The further constraints were set to default for Autodock Vina unless noted. Discovery studio 2019 program was used to visualize the best binding affinity of compounds. The reliability of the applied docking protocol was assessed by redocking cocrystal ligands into the active site of the protein domains. In addition, the positive control drug nintedanib exerts a therapeutic effect on PF. To make the conclusions more objective, we molecularly docked nintedanib with the potential core gene targets we screened.

### 2.5. Experimental Verification

#### 2.5.1. Animals and Experimental Design

Bleomycin (BLM) can induce the formation of a mouse model of PF, and mouse lungs have lung pathological changes similar to those of patients with PF. 75 male mice (18–22 g, 8 weeks old) were purchased from Beijing Vital River Laboratory Animal Technology Co., Ltd. (Beijing, China) and were adaptively reared for one week. After the mice were anesthetized, 15 mice in the control group were given normal saline via the oropharynx, and the rest of the mice were given BLM via the oropharynx to establish the PF model. The dose of BLM was 5 mg/kg. The BLM-induced PF model was randomized into four groups: BLM treatment group (model), BLM with WYJ high concentration treatment group (WYJ-H); BLM with WYJ medium concentration treatment group (WYJ-M), and BLM with WYJ low concentration treatment group (WYJ-L).

According to the previous literature and preliminary experimental results, WYJ-H, WYJ-M, and WYJ-L groups were given WYJ 1.2, 0.6, and 0.3 g/kg doses, respectively. The control group and the model group were treated with normal saline. Treatments were started 12 days after the establishment of the PF model, once a day, for 14 days. We sacrificed all mice on day 26 and stored lung tissue at −80°C for different experiments.

#### 2.5.2. Lung/Body Weight Ratio

We recorded the body weight (BW) and the whole lung weight (PW) after mice were sacrificed. Pulmonary indexes (PI) were calculated using the equation PI = PW/BW ∗ 100.

#### 2.5.3. Hydroxyproline Determination (HYP)

The lung tissue was processed according to the instructions of the Hydroxyproline Kit (Nanjing Jiancheng Institute of Bioengineering, Nanjing, China). HYP is the main marker of the development of PF. The level of HYP in the lung tissue was measured by the absorbance of 550 nm and the result was expressed with *μ*g/lung.

#### 2.5.4. Histopathological Examination

Lung tissues fixed with 10% formalin were embedded in paraffin and sectioned. Sections were stained with hematoxylin and eosin (H&E) and Masson (Sinopharm Chemical Reagent Beijing Co., Ltd., Beijing, China). Inflammation and collagen deposition of the sections were observed and assessed with an optical microscope.

#### 2.5.5. Culture of Human Pulmonary Fibroblast (HPF)

HPF is a normal human fibroblast cell line from the National Infrastructure of Cell Line Resource (Beijing, China). HPF cells were cultured in medium (DMEM) with 10% FBS and 1% PS and maintained at the cell incubator with 5% CO_2_ and 37°C. Cells were passaged at a confluence of 80%–90% using standard trypsinization techniques. HPF cells stimulated with TGF-*β*1 (10 ng/mL) were compared with cells that were grown in the same conditions but without stimulation of TGF-*β*1.

#### 2.5.6. Cell Viability Analysis

We assessed the viability of HPF cells pretreated with WYJ using Cell Counting Kit-8 (CCK-8, CK001, Lablead). HPF cells were seeded into a 96-well plate in the density of 2 × 10^4^ cells/well and treated with or without WYJ (0, 0.1, 1, 10, 20, or 50 *μ*g/mL). In each well, 200 *μ*L media was added with 10% CCK-8 solution after 48 h and then incubated at 37 for 1-2 h. The absorbance of the well was measured at 450 nm using a microplate reader (Molecular Devices, Sunnyvale, CA, USA).

#### 2.5.7. Quantitative Real-Time Polymerase Chain Reaction (qRT-PCR)

Total RNA extracted from HPF cells or mouse lung tissues using Trizol reagent (Qiagen, New York, USA) was purified with RNeasy column (Qiagen, New York, USA). The cDNA was obtained from purified RNA following the manufacturer's instructions of a reverse transcription kit (Qiagen, New York, USA). Full using QuantStudio6 Flex, gene expression analysis was performed following instructions of QuantiFast SYBR Green PCR kit (Qiagen, New York, USA). Fold changes of relative gene expression were calculated using the 2^−△△Ct^ method. Gene primer sequences are shown in Table 1.

#### 2.5.8. Western Blotting

Proteins obtained from lung tissue or cells were further analyzed with Bicinchoninic Acid (Applygen, Beijing, China) for protein concentration. Protein at 30–50 *μ*g/lanes was loaded and separated by SDS-PAGE gel electrophoresis. After SDS-PAGE, proteins were transferred to the polyvinylidene fluoride (PVDF) membranes (Millipore, Massachusetts, USA). Membranes were blocked with 5% fat-free milk dissolved with Tris-buffered saline with Tween 20 (TBST) for 120 min at room temperature. Rinsed with TBST, these membranes were incubated with primary antibodies at 4°C overnight. On the second day, blots were rinsed with TBST and then incubated with secondary antibody for 1 h. Blots were visualized by gel imager. GAPDH was used for loading positive control and the results were analyzed using Image J.

Antibodies: anti-*α*-SMA (ab5694, abeam, Cambridge, UK), anti-Collagen 1 (14695, Proteintech), anti-p-MAPK (ab201015, abeam), anti-MAPK (ab184699, abeam), anti-p-STAT3 (ab76315, abeam), anti-STAT3 (ab68153, abeam), anti-GAPDH (60004, Proteintech, Chicago, USA), anti-rabbit IgG (ab205718, abeam), and anti-mouse IgG (ab6728, abeam).

#### 2.5.9. Data and Statistical Analysis

One-way ANOVA was performed using GraphPad Prism software (version 8.0.2). *P* values <0.05 are adopted as statistically significant.

## 3. Results

### 3.1. Identified Ingredients of WYJ

**Figure 1:**
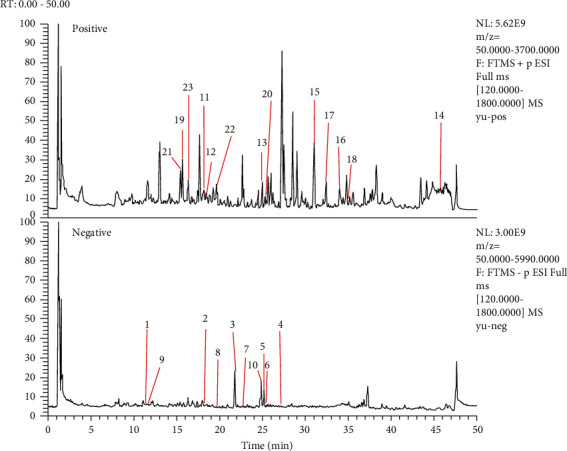
Total ion chromatogram.

Using UHPLC-LTQ-Orbitrap to detect and identify chemical compounds of WYJ (Figure 1), a total of 23 compounds were confirmed based on comprising with the published data. Information about the identified compounds is summarized in Tables 2 and 3.

### 3.2. Network and Pathway Analysis

**Figure 2:**
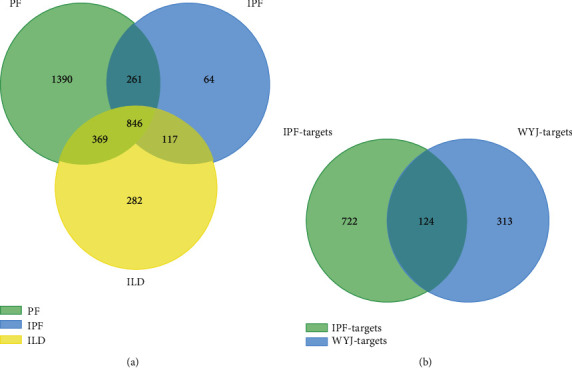
(a) Venn diagram of IPF-related targets. (b) Venn diagram of potential targets for WYJ treatment of PF.

Based on the network pharmacology methods above, 436 WYJ-related targets were obtained from SwissTargetPrediction and TCMSP after removing duplicate items. Information about WYJ-related targets is provided in Supplementary Table S1. Combining the results from GeneCards and OMIM database, a total of 2,867 targets for PF, 1,287 targets for IPF, and 1,613 targets for ILD were collected. As shown in Figure 2(a), 846 overlapping targets were critical targets for PF. The details are shown in Supplementary Table S2. Combining 463 WYJ-related targets with 846 PF-related targets, 124 targets could be potential targets for the WYJ treatment of PF (Figure 2(b)). Detailed information about 124 potential targets is provided in Supplementary Table S3.

**Figure 3:**
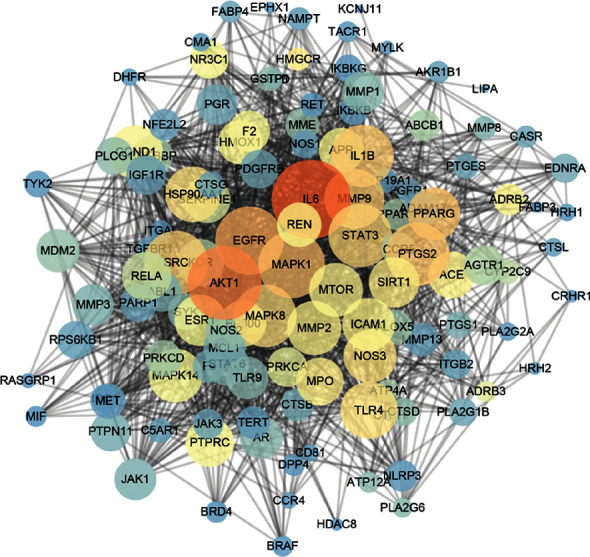
PPI network of potential targets.

STRING databases were used to explore the underlying interactions of 124 potential targets. There were 124 nodes and 1716 edges of the PPI network visualized by Cytoscape (Figure 3). According to the conditions of three topological (degree >22, bitterness centrality >0.00208, and, closeness centrality >0.5371179), 48 nodes were identified as the key targets of WYJ against PF (Supplementary Table S4).

**Figure 4:**
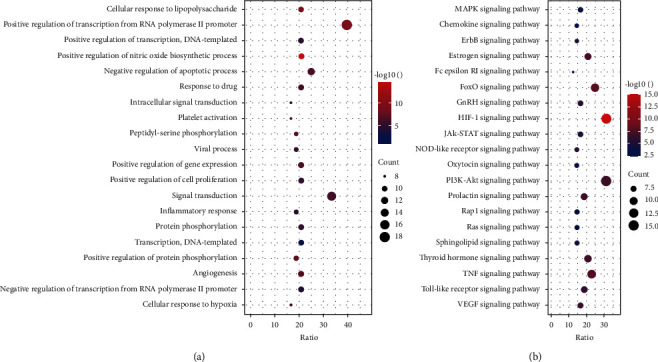
Bubble diagram of functional analysis. (a) Enrichment analysis of a biological process. (b) KEGG pathway enrichment analysis.

48 key targets obtained from PPI were selected to investigate the biological processes (BP) and mechanisms of WYJ on PF treatment. Results showed that BP were involved in PF treatment (Figure 4(a)), including positive regulation of transcription from RNA polymerase II promoter, inflammatory response, and signal transduction, etc. Concerning prediction of KEGG by David 6.8, the results revealed that most of the therapeutic targets are associated with signal transduction (e.g., MAPK signaling pathway and PI3K-protein kinase B (Akt) signaling pathway), inflammation (e.g., TNF signaling pathway and HIF-1 signaling pathway), and immune response (e.g., toll-like receptor signaling pathway and Jak-STAT signaling pathway). 20 significant KEGG pathway terms are shown in Figure 4(b).

**Figure 5:**
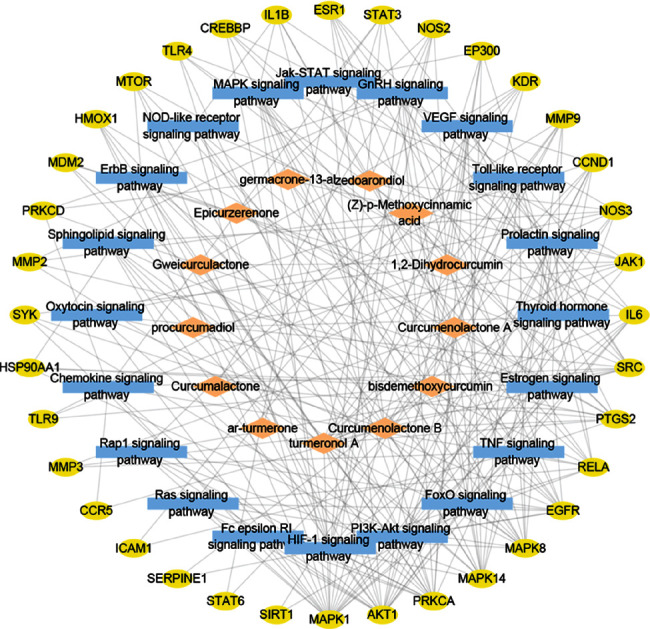
The integrated compound-target-pathway network visualized by Cytoscape.

Compound-target-pathway network explored the connection of compounds of WYJ, key targets, and 20 pathways. The integrated compound-target-pathway network comprised 69 nodes and 258 edges (Figure 5). STAT3, SRC, IL6, MAPK1, AKT1, EGFR, MAPK8, MAPK14, and IL1B were the nine most important nodes in the compound-target-pathway network comparing bitterness centrality, closeness centrality, and degree (Supplementary Table S5). 1,2-Dihydrocurcumin, ar-turmerone, bisdemethoxycurcumin, Curcumalactone, Curcumenolactone A, Curcumenolactone B, Epicurzerenone, germacrone-13-al, Gweicurculactone, (Z)-p-methoxycinnamic acid, procurcumadiol, turmeronol A, and zedoarondiol were the critical compounds.

### 3.3. Molecular Docking

**Figure 6:**
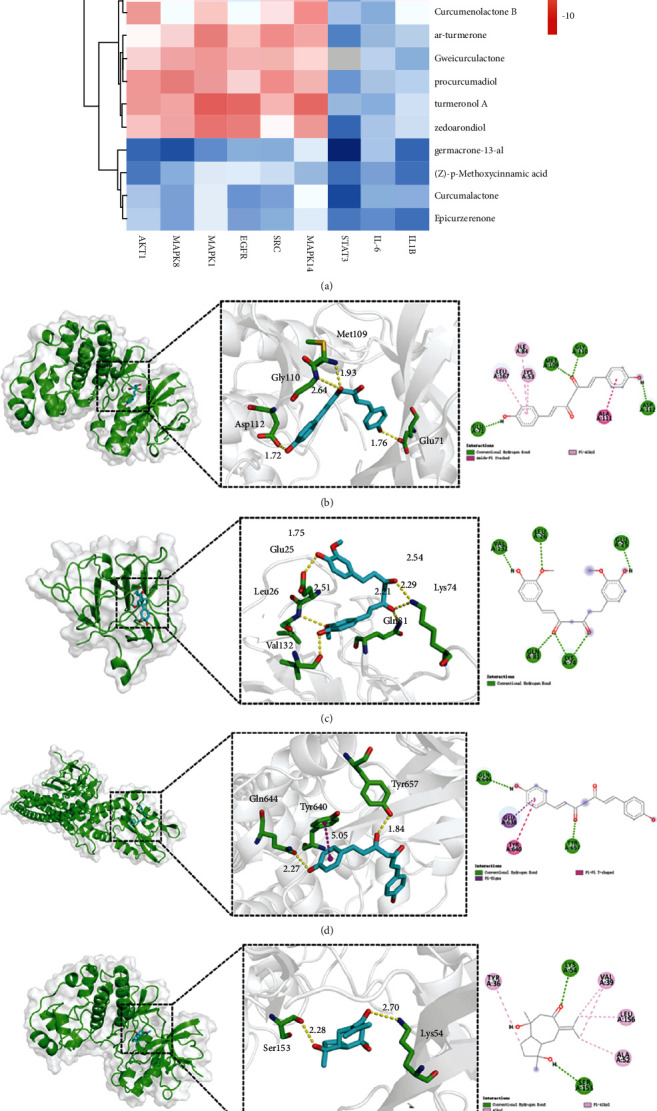
(a) Heat map of molecular docking. The redder the area, the lower the binding energy and the more stable the docking result. (b) Schematic diagram of the interplay between MAPK14 and bisdemethoxycurcumin. (c) Schematic diagram of the interplay between IL1B and 1,2-dihydrocurcumin. (d) Schematic diagram of the interplay between STAT3 and bisdemethoxycurcumin. (e) Schematic diagram of the interplay between MAPK1 and zedoarondiol.

RMSDs between the cocrystal structures and their original ligands were less than 2 Å, which indicated the method for docking was reasonable (Supplementary-Molecular Docking). We found that the affinity energies of most small molecules docking with proteins were less than −5.0 kcal/mol (Supplementary-Molecular Docking). Heat map of molecular docking is shown in the Figure 6(a). The major binding interactions between the compounds and target proteins include hydrophobic interaction and hydrogen bonding. For example (Figure 6(b)), bisdemethoxycurcumin formed four hydrogen bonds with amino acid residues Glu71, Met109, Gly110, and Asp112 with distances of 1.76 Å, 1.93 Å, 2.64 Å, and 1.72 Å, respectively, and formed hydrophobic interactions with amino acid residues Lys53, Ile84, Ala111, and Leu167.

### 3.4. Experimental Verification of WYJ Effects on the Pulmonary Fibrosis

#### 3.4.1. WYJ Alleviated BLM-Induced Pulmonary Fibrosis in Mice

**Figure 7:**
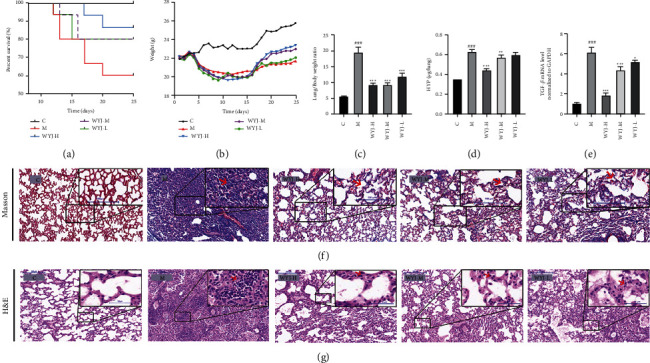
WYJ alleviated BLM-induced pulmonary fibrosis in mice. (a) Survival rate. (b) The body weight. (c) Lung/body weight ratio. (d) Hydroxyproline. (e) Lung tissue TGF-*β*1 mRNA. (f) The representative images of Masson staining. (g) The representative images of H&E staining. C, the control group. M, the model group. The datum was presented as meaning ± SEM (*n* ≥ 3). ^*∗*^*P* < 0.05, ^*∗∗*^*P* < 0.01 and ^*∗∗∗*^*P* < 0.001 versus the model group; ^#^*P* < 0.05, ^##^*P* < 0.01 and ^###^*P* < 0.001 versus the control group.

The BLM-induced PF model was used to verify the mechanism of WYJ on the treatment of PF. We observed significant decreases in survival rate, severe weight loss, and increase lung/body weight ratio in the BLM-induced PF model compared with the control group. The WYJ treatment increased the survival rate, attenuated weight loss, and reduced the lung/body weight ratio (Figures 7(a)–7(c)). HYP, an important fibrosis indicator of collagen deposition, increased in the model group and decreased by the WYJ treatment (Figure 7(d)). A large amount of TGF-*β*1 was observed during the development of PF [34, 35]. In our study, WYJ treatment downregulated the expression of TGF-*β*1 mRNA in lung tissue of the PF model (Figure 7(e)). As revealed by Masson staining (Figure 7(f)) and H&E staining (Figure 7(g)), WYJ treatment decreased the infiltration of inflammatory cells and relieved thrombus and structure destruction compared with the model group. The above results confirmed that WYJ exerted a therapeutic effect on PF.

**Figure 8:**
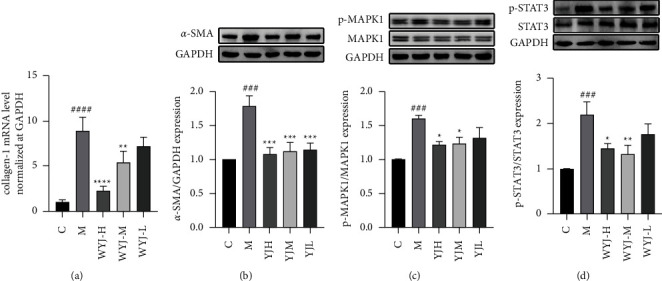
The effect of WYJ on BLM-induced pulmonary fibrosis mice. (a) mRNA expression of collagen I in lung tissues was measured by qRT-PCR. (b–d) The relative protein levels of *α*-SMA, p-MAPK1, and p-STAT3. C, the control group. M, the model group. The datum was presented as meaning ± SEM (*n* ≥ 3). ^*∗*^*P* < 0.05, ^*∗∗*^*P* < 0.01 and ^*∗∗∗*^*P* < 0.001 versus the model group; ^#^*P* < 0.05, ^##^*P* < 0.01 and ^###^*P* < 0.001 versus the control group.

Excessive deposition of ECM, such as collagen I [36], is characteristic of fibrosis. Compared with the model group, the WYJ treatment suppressed the mRNA expression of collagen I (Figure 8(a)). The differentiation of lung fibroblasts into myofibroblasts is essential for the development of PF [37, 38], and *α*-SMA is a marker of myofibroblasts [35]. The WB results indicated that the expression of *α*-SMA decreased after WYJ treatment compared with the model group (Figure 8(b)), indicating that WYJ reduced the production of myofibroblasts.

According to the results of network pharmacology analysis, it is worth noting that STAT3 is the most important target in the compound-target-pathway network. Moreover, IL6, MAPK1, AKT1, EGFR, MAPK8, MAPK14, and IL1B were the key targets interacting closely with the MAPK signaling pathway. Therefore, we speculated that WYJ might improve PF mainly via the STAT3 and MAPK signaling pathways. Abundant evidence has indicated that STAT3 plays a key role in the occurrence and development of fibrosis [39, 40], whereas the conduction of the MAPK signaling pathway is closely related to PF [41, 42]. Therefore, we experimentally verified the expression of key proteins in these two pathways by WB. Compared with the control group, the phosphorylation levels of MAPK1 increased, while this trend was markedly reduced after WYJ treatment (Figure 8(c)). In addition, the same manner was observed in the expression level of phosphorylated STAT3 (Figure 8(d)). Those results revealed that the protective effect of WYJ on PF might be related to the inhibition of MAPK and STAT3 pathways.

#### 3.4.2. WYJ Inhibited Fibroblast Activation in In Vitro Experiments

**Figure 9:**
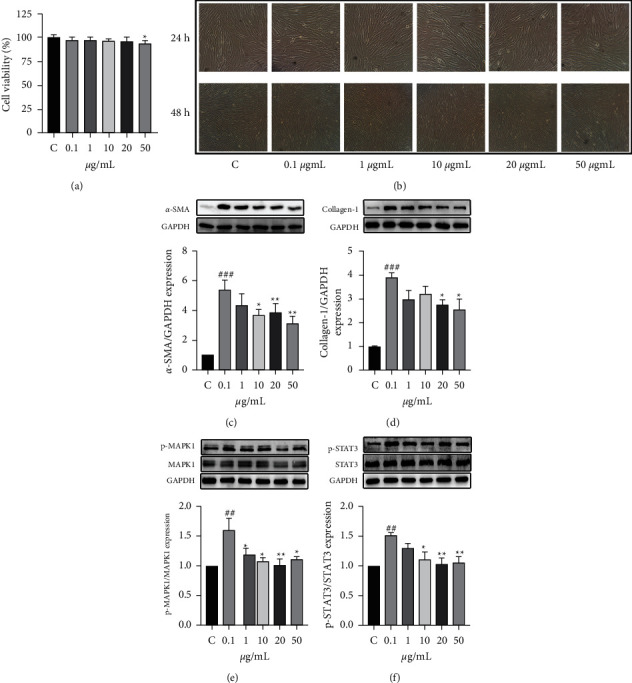
(a, b) The effect of WYJ on HPF activity. (c–f) The relative protein levels of *α*-SMA, collagen I, p-MAPK1, and p-STAT3. C, the control group. M, the model group. The datum was presented as meaning ± SEM (*n* ≥ 3). ^*∗*^*P* < 0.05, ^*∗∗*^*P* < 0.01 and ^*∗∗∗*^*P* < 0.001 versus the model group; ^#^*P* < 0.05, ^##^*P* < 0.01, and ^###^*P* < 0.001 versus the control group.

In vitro experiments further verified the antipulmonary fibrosis effect of WYJ. We observed no change in cell morphology at different concentrations of WYJ using microscopy. However, CCK-8 results showed that, at a concentration of 50 *μ*g/mL, the viability of HPF cells decreased slightly (Figures 9(a) and 9(b)). Therefore, we used 0.1–20 *μ*g/mLWYJ to observe the effect of WYJ on TGF-induced HPF cells.

Activated lung fibroblasts, the primary roles in the progress of fibrogenesis, express excessive *α*-SMA and collagen I. We used TGF-*β*1 to promote fibroblast activation [35] and evaluated the expression of *α*-SMA and collagen I after WYJ treatment. The results demonstrated that HPF cells stimulated with TGF-*β*1 significantly increased the production of *α*-SMA and collagen I protein. HPF cells pretreated with WYJ markedly decreased expression of *α*-SMA and collagen I protein (Figures 9(c) and 9(d)). And the results also showed that WYJ prevented the activation of fibroblasts by downregulating p-MAPK1 and p-STAT3 in vitro (Figures 9(e) and 9(f)).

## 4. Discussion and Conclusions

PF is a chronic progressive tissue repair response that can lead to irreversible scarring and remodeling of the lungs [9]. As mentioned in the introduction section, the occurrence of PF involves lung injury, the production of profibrotic factors, and the deposition of ECM. Reducing the expression of profibrotic factors and the production of ECM is extremely important for alleviating PF. Pirfenidone and nintedanib, approved by the FDA, have side effects and withdrawal symptoms [11, 13–14], and there is an urgent need to develop drugs with fewer side effects. TCM has accumulated valuable information alleviating lung diseases. WYJ, from steamed roots of Curcuma beauty, was included in the 2020 edition of the Pharmacopoeia of People's Republic of China. Modern studies have shown that the ingredients of WYJ can alleviate diseases through antibacterial, antitumor, anti-inflammatory, and antioxidant effects [16, 17]. Among the ingredients of WYJ, bisdemethoxycurcumin can reduce renal fibrosis through anti-inflammatory, antioxidant, and antiapoptotic effects [43, 44], ar-turmerone plays an antipsoriatic effect by inhibiting cell proliferation and reducing the expression of inflammatory cytokines [45], p-Methoxycinnamic exerts anticancer properties through anti-inflammatory effects [46], and curcumin has anti-pulmonary fibrosis effect in a murine model [47, 48]. Based on the above research, we studied the impact of WYJ on PF and related molecular mechanisms.

It has developed many animal models to study the pathogenesis and treatment of different diseases in humans. The BLM-induced PF mouse model has been widely accepted as a model for studying PF. This model could cause a pulmonary pathological change, which is like that of patients with lung fibrosis [49, 50]. Compared with the model group, we observed through pathological slices that WYJ treatment could decrease the infiltration of inflammatory cells and relieve thrombus and structure destruction of lung tissues (Figures 7(f) and 7(g)). Previous studies have indicated that TGF-*β*1, *α*-SMA, and ECM proteins (such as fibronectin, collagen I, and collagen III), and HYP are the main markers of the development of PF [7–10, 51]. Our results showed that WYJ significantly inhibits the expression of these fibrosis markers in PF mice, which suggests that WYJ has an antifibrosis effect in the lungs. The weight change and survival curve also showed that WYJ can improve the quality of life of PF mice.

Various lung cell types (such as alveolar epithelial cells, endothelial cells, mesenchyme fibroblasts, and circulating fibroblasts) are involved in the occurrence and development of PF under pathological conditions [52]. However, differentiation of lung fibroblasts into myofibroblasts is essential for the development of PF [37, 38]. As the most critical fibrosis-promoting factor, TGF-*β*1 can induce lung fibroblasts to differentiate into myofibroblasts [7]. Therefore, we used TGF-*β*1 to induce HPF and observed whether WYJ can inhibit HPF proliferation and differentiation. We found that WYJ significantly reduced the expression of fibrosis markers (*α*-SMA, collagen I) in HPF cells induced by TGF-*β*1. These results are consistent with the results of the mouse model.

Multiple targets and multiple pathways regulate the occurrence of PF. So, only targeting one target for treatment will not eliminate the pathological activation of other targets in PF. Therefore, we used network pharmacology to screen out 9 critical targets of WYJ in the treatment of PF. MAPK1 (ERK2), MAPK8 (JNK1), and MAPK14 (p38-*α*) are essential members of the MAPKs family. MAPKs signaling cascades regulate processes, including cell cycle progression, cell migration, cell survival, and differentiation [53]. More and more evidence showed that the MAPK pathway is involved in many aspects of PF, such as the recruitment of fibroblasts and the deposition of extracellular matrix [41, 42, 54, 55]. The production of IL-1*β* is achieved through a two-step process. The activation of the MAPK pathway participates in the synthesis of the inactive 31 kDa precursor (pro-IL-1*β*), and the inflammation induces the production of the active/mature form of IL-1*β* (17 kDa) [56, 57]. IL-1*β* not only is an effective inducer of TGF-*β*1 [58] but also causes BLM-induced pulmonary toxicity through the caspase-1/IL-1*β* pathway [59]. EGFR is a transmembrane protein with intrinsic tyrosine kinase activity. Studies showed that EGFR is abnormally activated in animal models of PF and mediates the pathogenesis of pulmonary fibrosis [60–62]. Inhibition of EGFR reduced the phosphorylation of ERK1/2, leading to inhibition of the activation of profibrotic pathways [63]. KEGG analysis results showed that IL6, MAPK1, AKT1, EGFR, MAPK8, MAPK14, and IL1B were the key targets interacting closely with the MAPK signaling pathway. IL6, SRC, and ERGF can cause the activation of the downstream target STAT3, leading to lung fibrosis. STAT3 regulates cell growth, proliferation, differentiation, and migration [64]. Phosphorylated STAT3 has been reported to be involved in PF [39, 40, 65, 66]. The primary receptor coupled with STAT3 is the gp130/IL-6 receptor family, including IL-6 [67, 68]. In the non-canonical TGF*β* signaling, EGFR and SRC also mediate the phosphorylation and activation of STAT3 [69–72]. IL6, SRC, and ERGF can cause the activation of the downstream target STAT3, leading to lung fibrosis. To sum up, we speculated that WYJ might improve PF mainly via the MAPK and STAT3 signaling pathways. We performed experimental verification of the expression of essential proteins (MAPK1 and STAT3) in the two pathways. Western blot results showed that the phosphorylation of MAPK1 and STAT3 in the lung tissues of the model group was significantly increased, which is consistent with previous studies [18, 42, 63, 73]. WYJ alleviated PF by reducing the phosphorylation of MAPK1 and STAT3. In in vitro experiments, we also observed the same results, confirming the reliability of in vivo experiments.

The molecular docking results described above are helpful for a basic understanding of the mechanism of drug action. If the affinity energy is less than −5 kcal/mol, it indicates that the target has certain binding activity with the compound. We found that the affinity energies of most small molecules docking with proteins were less than −5.0 kcal/mol. We performed further analysis of the interaction between the target proteins and the compounds. The comparative analysis showed that positive control drug nintedanib showed good binding activity, but some of the active compounds of WYJ (bisdemethoxycurcumin, 1,2-dihydrocurcumin) showed higher binding activity with the core gene target to some extent, which also provides direction for the development of new drugs and subsequent research.

In conclusion, our study identified that WYJ alleviated pulmonary fibrosis. According to the results of network pharmacology and molecular docking, the pharmacological mechanism of WYJ in the treatment of PF was further validated by experiment. The inhibitory effect of WYJ on PF may function mainly by inhibiting MAPK and STAT3 signaling pathways, which provide new insights for the treatment of WYJ at PF. The method of exploring the pharmacological mechanism of WYJ used in our study provides a novel approach to explain the pharmacological basis of other herbs. Network pharmacology has limitations. More experiments are needed to verify the reliability anti-fibrotic effect of WYJ.

## Figures and Tables

**Table 1:** Gene primer sequences.

Gene		Primer (5′-3′)	Accession no.
Mus-GAPDH	Forward	AGGTTGTCTCCTGCGACTTCA	NM_008084.3
	Reverse	TGGTCCAGGGTTTCTTACTCC	
Mus-col1a1	Forward	CCAAGAAGACATCCCTGAAGTCA	NM_007742.4
	Reverse	TGCACGTCATCGCACACA	
Mus-TGF-*β*1	Forward	ATGACATGAACCGGCCCTT	NM_011577.2
	Reverse	AGTTGGTATCCAGGGCTCTCC	

**Table 2:** Chemical compounds of WYJ in negative ion mode.

No.	TR (min)	Molecular formula	Accurate mass [M–H]−	Calculated mass [M–H]−	Fragmentation (MS/MS)	Error (bpm)	Identified compounds	PubChem CID
1	11.58	C10H10O3	177.0552	177.0555	162.0325; 113.002; 92.9957	1.694	(Z)-p-methoxycinnamic acid	1550936
2	18.13	C15H18O3	245.1178	245.1186	180.9893; 175.0763; 57.0345	3.264	Curcolone	12304272
3	21.68	C15H18O3	245.1178	245.1182	224.9971; 201.1285; 180.9894	1.632	Editors	46173920
4	27.14	C15H20O3	247.1334	247.1339	229.1234; 203.1078; 189.0921; 161.0971	2.023	Curcumenolactone A	10083354
5	25.28	C15H22O3	249.1491	249.1497	228.9897; 205.1598; 154.9738; 112.9857	2.408	Procurcumadiol	14633012
6	25.29	C15H24O3	251.1647	251.1654	207.1753; 191.1442; 125.0972; 57.0346	2.787	Zedoarondiol	24834047
7	22.62	C15H22O4	265.144	265.1445	194.9886; 112.9856; 68.9957	1.886	Zedoalactone A	15226639
8	19.77	C15H12O5	271.0607	271.0613	186.9991; 151.0036; 119.0501	2.214	Naringenin	439246
9	11.56	C15H20O5	279.1233	279.1236	235.1339; 217.1232; 181.0869; 137.097	1.075	Zedoalactone B	15226640
10	24.88	C21H22O6	369.1338	369.1347	354.1110; 247.0974; 218.0585; 140.0114	2.438	1,2-Dihydrocurcumin	5372374

**Table 3:** Chemical compounds of WYJ in negative ion mode.

No.	TR (min)	Molecular formula	Accurate mass [M + H]+	Calculated mass [M +H]+	Fragmentation (MS/MS)	Error (bpm)	Identified compounds	PubChem CID
11	17.67	C10H12O	149.0966	149.0962	121.0649	−2.683	Cuminal	326
12	18.02	C19H16O4	309.1127	309.1111	281.0920; 62.9826	−5.176	Bisdemethoxycurcumin	45934475
13	24.07	C15H20	201.1643	201.1638	169.0860; 141.091; 113.0601	−2.486	Calacorene	12302243
14	45.72	C15H22	203.18	203.1796	175.1482; 133.1013; 109.1016; 81.0706	−1.969	(+)-alpha-curcumene	3083834
15	30.43	C15H16O	213.1279	213.1275	195.1169; 157.1013	−1.877	Pyrocurzerenone	12314812
16	32.49	C15H20O	217.1592	217.1588	161.0961; 137.0961; 95.0860;	−1.842	Ar-turmerone	160512
17	31	C15H16O2	229.1228	229.1223	NA	−2.182	Gweicurculactone	130117
18	34.07	C15H18O2	231.1385	231.1381	213.1275; 173.0962; 85.0653	−1.731	Epicurzerenone	5317062
19	15.7	C15H20O2	233.1541	233.1536	215.1432; 187.1482; 175.1118	−2.145	Furanogermenone	6439596
20	24.49	C15H20O2	233.1541	233.154	NA	−0.429	Turnaround A	15858385
21	15.39	C15H22O2	235.1698	235.1692	217.1587; 189.1638; 135.1169; 97.0653	−2.551	Germacrone-13-al	46173921
22	19.31	C15H24O2	237.1854	237.1847	219.1381; 173.1362; 133.1013	−2.951	Circumstance	15071433
23	16.39	C15H20O3	249.149	249.1485	231.1379	−2.007	Curcumenolactone B	10264037

## Data Availability

The datasets used and analyzed during the current study are available from the corresponding author on reasonable request.

## Supplementary Materials

Supplementary Materials Supplementary Table S1: information about WYJ-related targets. Supplementary Table S2: information about PF-related targets. Supplementary Table S3: targets information at the intersection of drug targets and disease targets. Supplementary Table S4: 48 key targets of WYJ against PF. Supplementary Table S5: core gene information filtered according to the “betweenness,” “closeness,” and “degree” values. Supplementary-Molecular Docking: information about molecular docking.
