# Supplementary material for: Network Pharmacology Prediction and Molecular Docking-Based Strategy to Discover the Potential Pharmacological Mechanism of Wen-Yu-Jin against Pulmonary Fibrosis in a Mouse Model
Source: Evid Based Complement Alternat Med. 2022 Feb 10;2022:7753508. doi: 10.1155/2022/7753508 (PMC8853792; doi:10.1155/2022/7753508)
Supplement: Supplementary Materials — Supplementary Table S1: information about WYJ-related targets. Supplementary Table S2: information about PF-related targets. Supplementary Table S3: targets information at the intersection of drug targets and disease targets. Supplementary Table S4: 48 key targets of WYJ against PF. Supplementary Table S5: core gene information filtered according to the “betweenness,” “closeness,” and “degree” values. Supplementary-Molecular Docking: information about molecular docking. [file 7753508.f1.zip › 7753508.f1/Supplementary- Molecular Docking.pdf]

Target	Uniprot ID	PDBID	Original Ligand	x	y	z	Grid box
STAT3	P40763	6NJS	KQV	13.498	54.118	0.101	53*81*58
SRC	P12931	4XMO	46G	23.151	32.021	57.200	53*55*43
IL-6	P05231	1ALU	-	-0.315	-19.932	8.838	48*50*40
MAPK1	P28482	4QTA	38Z	27.873	54.529	35.163	57*81*44
AKT1	P31749	4GV1	0XZ	-20.286	3.748	11.736	50*49*52
EGFR	P00533	3POZ	03P	18.746	31.832	11.626	55*49*56
MAPK8	P45983	4L7F	1V5	39.787	1.607	73.990	34*49*40
MAPK14	Q16539	3ROC	29A	35.463	38.806	22.632	45*38*70
IL1B	P01584	5R8Q	JGY	39.787	1.607	73.990	34*49*40

Target	PDBID	References
STAT3	6NJS	https://www.rcsb.org/structure/6NJS
SRC	4XMO	https://www.rcsb.org/structure/4XMO
IL-6	1ALU	https://www.rcsb.org/structure/1ALU
MAPK1	4QTA	https://www.rcsb.org/structure/4QTA
AKT1	4GV1	https://www.rcsb.org/structure/4GV1
EGFR	3POZ	https://www.rcsb.org/structure/3POZ
MAPK8	4L7F	https://www.rcsb.org/structure/4L7F
MAPK14	3ROC	https://www.rcsb.org/structure/3ROC
IL1B	5R8Q	https://www.rcsb.org/structure/5R8Q

Prior to docking the ligands against the target proteins, the redocking of the co-crystal structures and their original ligands was carried out. All of the RMSDs was less than 2 Å which indicated the method for docking was reasonable.

Target	RMSD	Overlay of the docking result (green) and original ligand (blue)		Target	RMSD	Overlay of the docking result (green) and original ligand (blue)
STAT3	1.7302	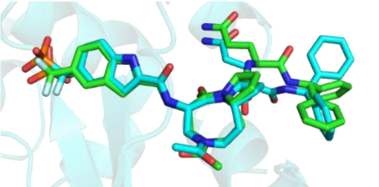		MAPK1	1.2972	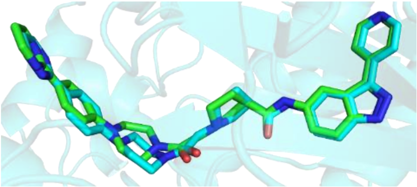
SRC	0.3809	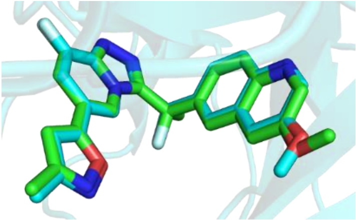		AKT1	1.2037	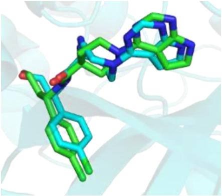
MAPK8	1.263	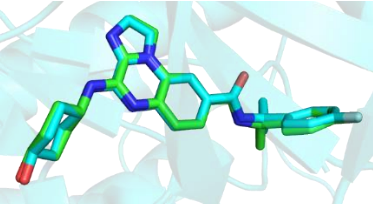		EGFR	1.5066	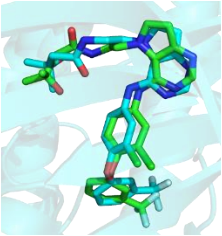
MAPK14	1.6399	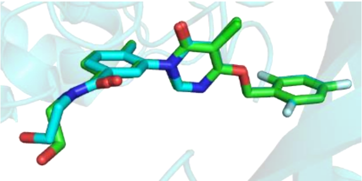		IL1B	0.1241	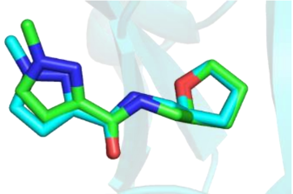
IL-6	-	-				

The results of different affinity energy and Heat map of molecular docking

Ligand	STAT3	SRC	IL-6	MAPK1	AKT1	EGFR	MAPK8	MAPK14	IL1B
Gweicurculactone	-4.6	-7.7	-6.4	-8.1	-7.4	-7.7	-7.9	-7.3	-5.9
Ar-turmerone	-5.1	-8.2	-6.0	-8.4	-7.0	-7.6	-7.3	-7.9	-6.3
Curcumalactone	-3.9	-5.7	-5.9	-6.7	-6.2	-5.5	-5.7	-6.9	-5.8
(Z)-p-Methoxycinnamic acid	-4.8	-6.5	-5.6	-6.7	-5.0	-6.7	-5.8	-6.0	-4.8
bisdemethoxycurcumin	-7.4	-10.1	-6.4	-10.3	-8.8	-9.2	-10.3	-10.8	-6.9
Epicurzerenone	-5.0	-5.9	-5.4	-6.7	-6.3	-5.6	-5.6	-6.7	-4.8
Curcumenolactone A	-5.6	-7.4	-6.2	-7.4	-7.0	-6.7	-7.1	-7.7	-7.1
Curcumenolactone B	-6.2	-7.2	-5.9	-7.5	-8.1	-6.9	-6.8	-8.2	-6.8
1,2-Dihydrocurcumin	-9.9	-9.0	-6.0	-9.9	-10.6	-9.3	-9.7	-8.6	-5.7
Turmeronol A	-6.0	-7.7	-5.8	-8.9	-8.1	-8.7	-7.8	-8.8	-6.6
Zedoarondiol	-4.6	-7.1	-6.2	-8.6	-7.5	-8.4	-7.8	-8.1	-6.6
Germacrone-13-al	-2.7	-5.8	-6.1	-5.3	-4.7	-5.8	-4.2	-6.7	-4.7
Procurcumadiol	-5.5	-8.2	-6.1	-8.0	-8.0	-7.3	-8.4	-7.7	-6.4
Nintedanib	-10.3	-8.4	-8.0	-10.6	-9.9	-10.1	-11.2	-9.5	-7.6

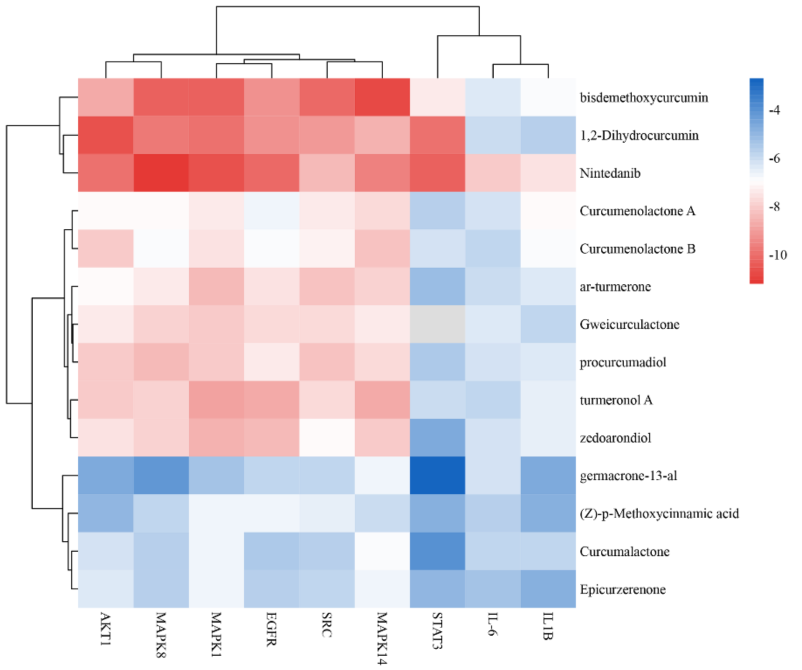

Residues participating in the protein-ligand interactions

Target	Ligand	Interactions	2D diagram
	Ar-turmerone	Hydrogen Bond: Tyr640 (1)<br>Hydrophobic: Met648, Ile653, Lys658	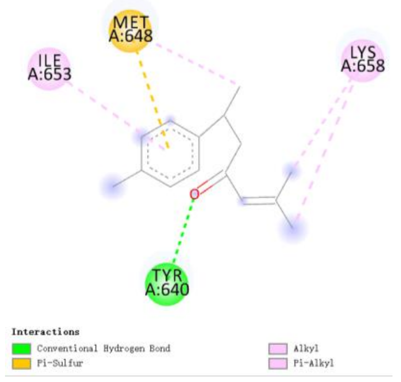
STAT3	Bisdemethoxycurcumin	Hydrogen Bond: Gln644, Tyr657 (2)<br>Hydrophobic: Glu638, Tyr640	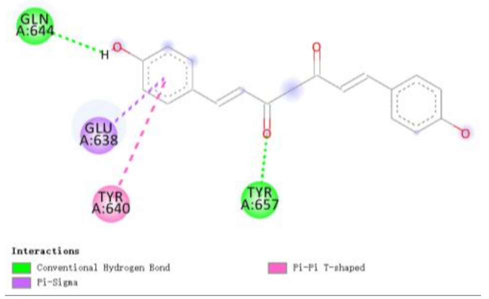
	Curcumenolactone B	Hydrogen Bond: Ser611, Ser613, Glu638 (3)<br>Hydrophobic: Pro639	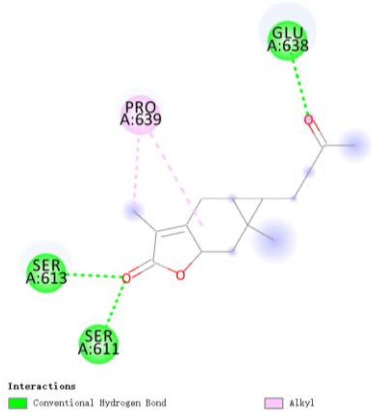

	1,2-Dihydrocurcumin	Hydrogen Bond: Lys615, Glu616, Gln644 (3)<br>Hydrophobic: Glu638, Tyr640	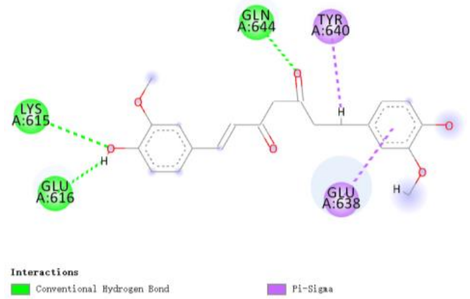
	Turmeronol A	Hydrogen Bond: Glu638, Gln644 (2)<br>Hydrophobic: Val637, Tyr640	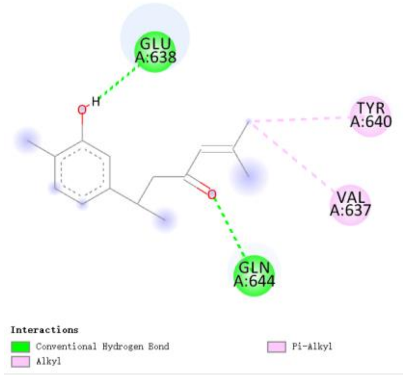
SRC	Gweicurculactone	Hydrophobic: Ile1084, Val1092, Ala1108, Leu1140, Leu1157, Tyr1159, Met1211. Tyr1230	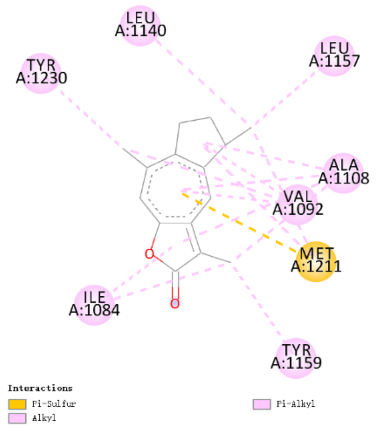

	(Z)-p-Methoxycinnamic acid	Hydrogen Bond: Met1160, Met1211, Asp1222 (3)<br>Hydrophobic: Val1092, Ala1108, Leu1157, Met1211	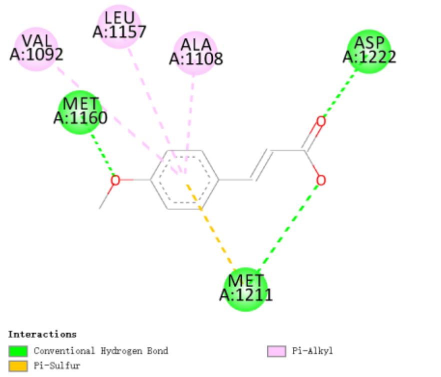
	Bisdemethoxycurcumin	Hydrogen Bond: Ile1084, Arg1208 (2)<br>Hydrophobic: Ile1084, Tyr1159, Mer1211, Tyr1230	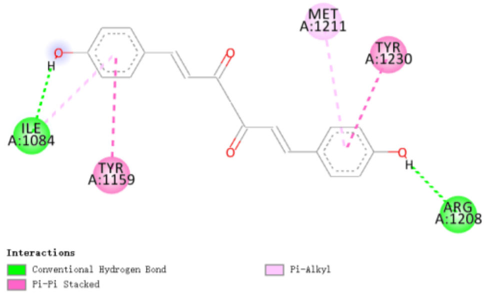
	1,2-Dihydrocurcumin	Hydrogen Bond: Met1160, Asp1222 (3)<br>Hydrophobic: Val1092, Ala1108, Met1211, Tyr1230	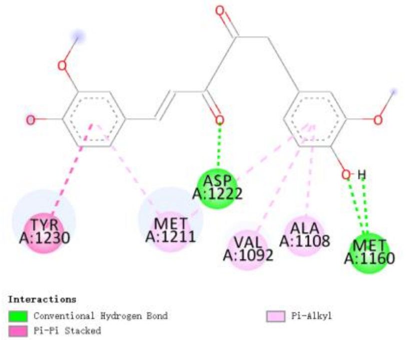

	Procurcumadiol	Hydrogen Bond: Arg1086, Tyr1230 (2)<br>Hydrophobic: Ile1084, Val1092, Met1211, Ala1226, Tyr1230	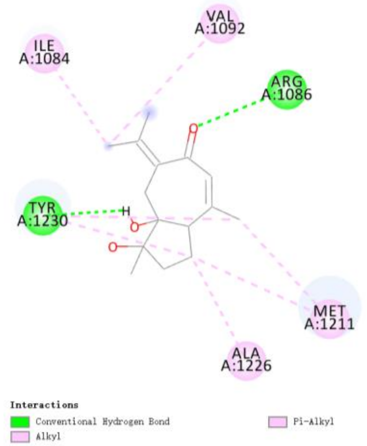
IL6	Ar-turmerone	Hydrogen Bond: Arg179 (1)<br>Hydrophobic: Arg30, Leu33, Leu178	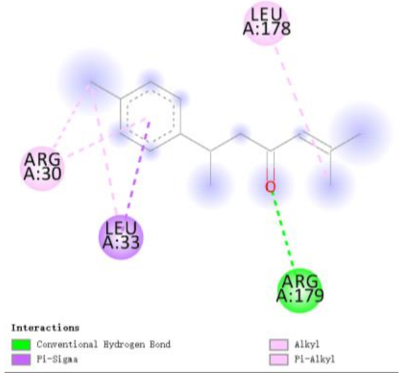
	Curcumalactone	Hydrogen Bond: Leu64 (1)<br>Hydrophobic: Leu62, Leu64, Lys66, Leu165	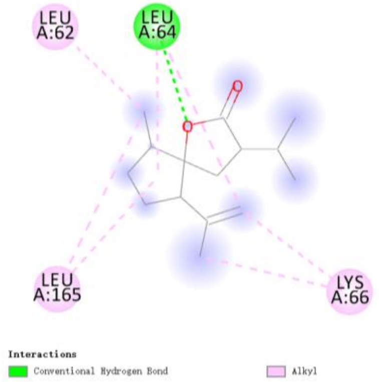

	Bisdemethoxycurcumin	Hydrogen Bond: Ser107, Asp160 (2)<br>Hydrophobic: Lys46, Arg104, Phe105	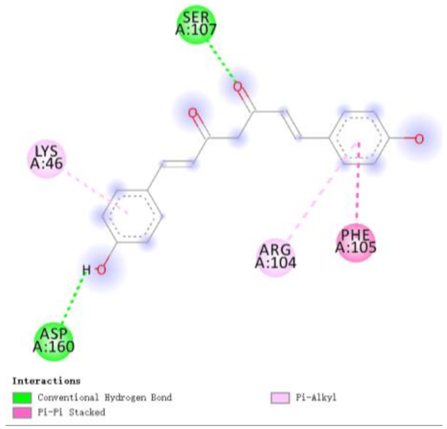
	Curcumenolactone A	Hydrogen Bond: Arg104, Gln156 (2)<br>Hydrophobic: Phe105	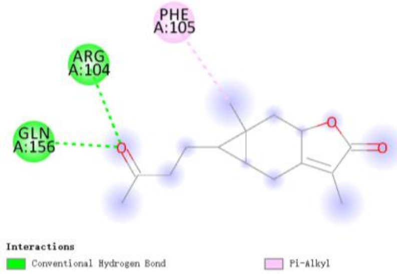
	Germacrone-13-al	Hydrogen Bond: Thr43, Glu106 (2)	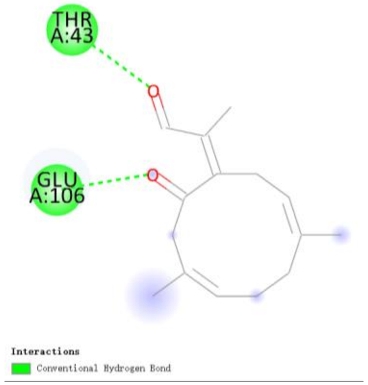

<table>
<tr><td rowspan="3">MAPK1</td><td>Gweicurculactone</td><td>Hydrogen Bond: -
Hydrophobic: Ala35, Tyr36, Ile56, Tyr64, Arg67</td><td>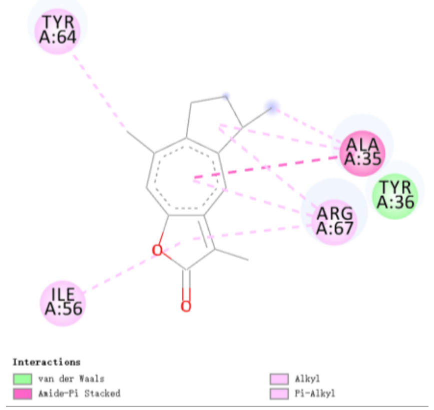
</td></tr>
<tr><td>Bisdemethoxycurcumin</td><td>Hydrogen Bond: Lys54, Met108 (3)
Hydrophobic: Val39, Ala52, Lys54, Ile56, Glu71, Leu156</td><td>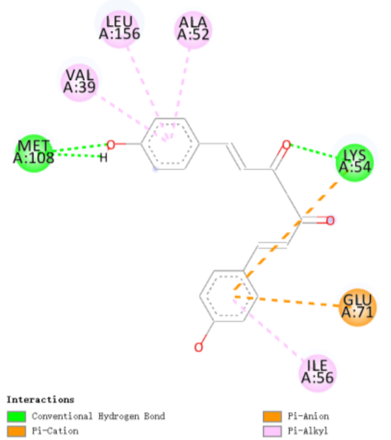
</td></tr>
<tr><td>Epicurzerenone</td><td>Hydrogen Bond: Lys54 (1)
Hydrophobic: Tyr36, Val39, Ala52, Lys54, Ile84, Leu156, Cys166</td><td>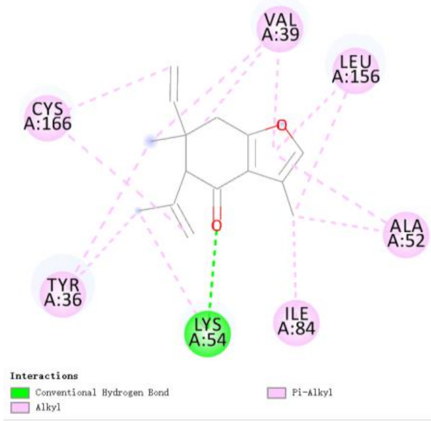
</td></tr>
</table>

	1,2-Dihydrocurcumin	Hydrogen Bond: Lys54, Arg67, Asp106, Met108 (5)<br>Hydrophobic: Val39, Ala52, Arg67, Leu156	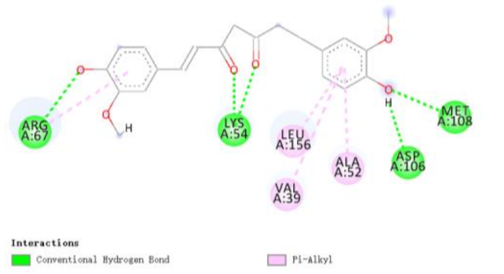
	Zedoarondiol	Hydrogen Bond: Lys54, Ser153 (2)<br>Hydrophobic: Tyr36, Val39, Val52, Leu156	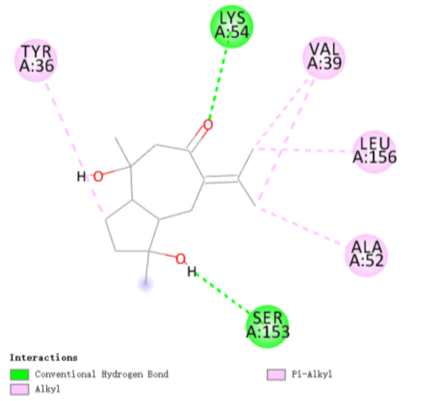
AKT1	Bisdemethoxycurcumin	Hydrogen Bond: Glu191, Glu228, Ala230 (3)<br>Hydrophobic: Val164, Ala177, Met227, Met281, Asp292	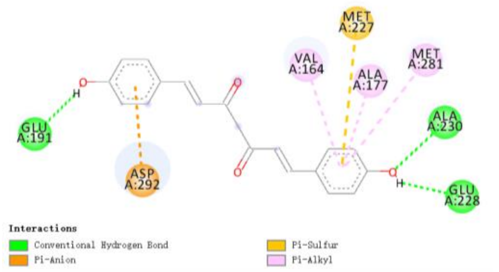
	Curcumenolactone A	Hydrogen Bond: Asp439 (1)<br>Hydrophobic: Val164, Met281	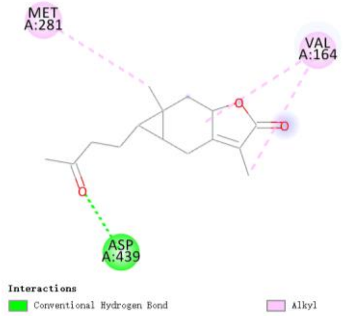

<table>
<tr><td></td><td>1,2-Dihydrocurcumin</td><td>Hydrogen Bond: Glu191, Glu228 (2)
Hydrophobic: Phe161, Val164, Ala177, Lys179, Met281, Asp292</td><td>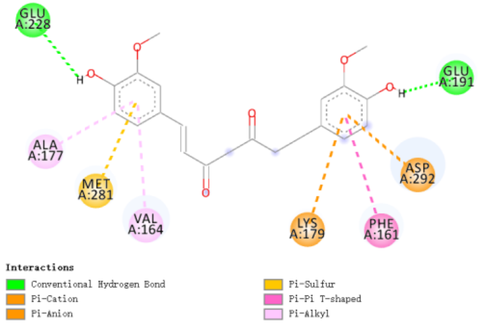
</td></tr>
<tr><td></td><td>Turmeronol A</td><td>Hydrogen Bond: Glu228 (1)
Hydrophobic: Leu156, Val164, Ala177, Mer227, Met281, Phe438, Phe442</td><td>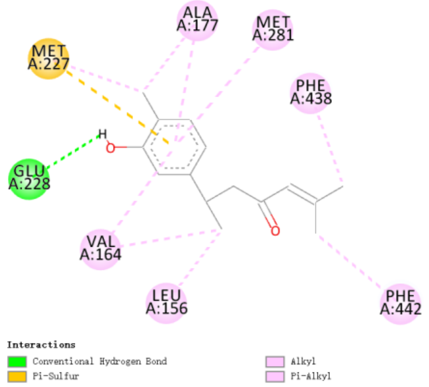
</td></tr>
<tr><td></td><td>Procurcumadiol</td><td>Hydrogen Bond: Lys158, Asp292 (2)
Hydrophobic: Leu156, Val164, Phe438</td><td>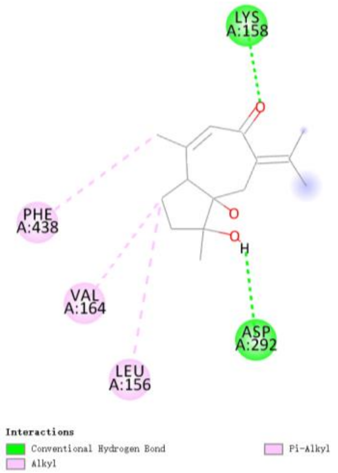
</td></tr>
</table>

EGFR	Ar-turmerone	Hydrogen Bond: Thr854 (1)<br>Hydrophobic: Val726, Ala743, Lys745, Met766, Leu788, Leu844, Phe856	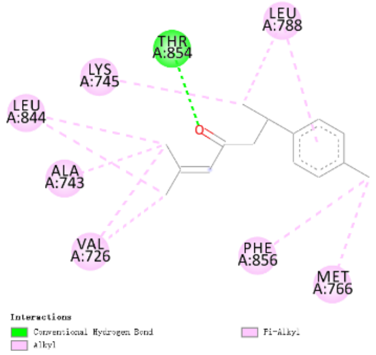
	(Z)-p-Methoxycinnamic acid	Hydrogen Bond: Asp855 (1)<br>Hydrophobic: Lys745, Leu788	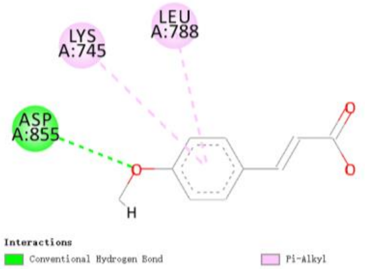
	Bisdemethoxycurcumin	Hydrogen Bond: Met766, Cys775, Met793, Thr854 (4)<br>Hydrophobic: Leu718, Ala743, Met766, Leu777, Leu844	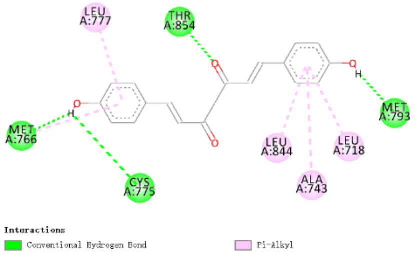

	1,2-Dihydrocurcumin	Hydrogen Bond: Lys745, Thr854, Phe856 (3)<br>Hydrophobic: Leu718, Val726, Met766, Leu788, Phe856, Leu858	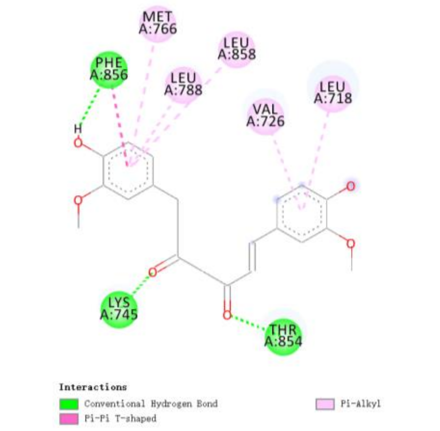
	Zedoarondiol	Hydrogen Bond: Thr854 (1)<br>Hydrophobic: Leu718, Lys745, Leu844	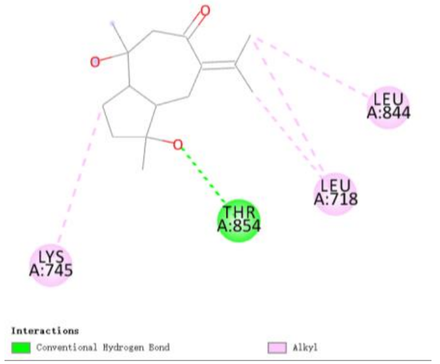
MAPK8	Gweicurculactone	Hydrogen Bond: Met111 (1)<br>Hydrophobic: Ile32, Val40, Ala53, Lys55, Met108, Leu110, Val158, Leu168	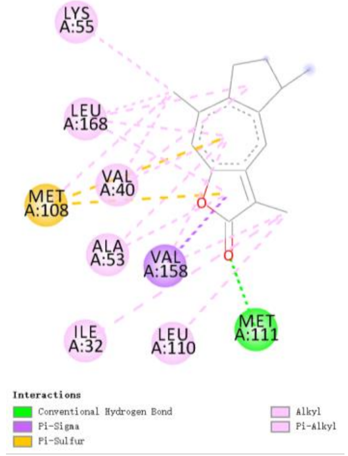

<table>
<tr><td></td><td>Ar-turmerone</td><td>Hydrogen Bond: Lys55 (1)
Hydrophobic: Ile32, Val40, Ala53, Lys55, Leu57, Leu168</td><td>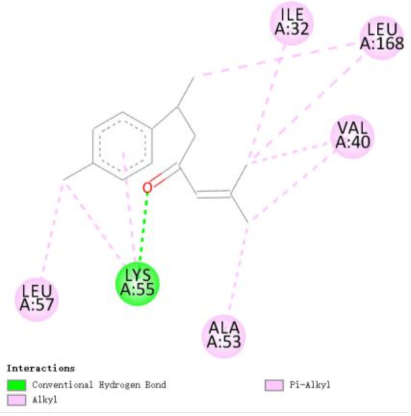
</td></tr>
<tr><td></td><td>Bisdemethoxycurcumin</td><td>Hydrogen Bond: Lys55, Glu109, Asn114 (3)
Hydrophobic: Ala53, Met108, Val158, Leu168</td><td>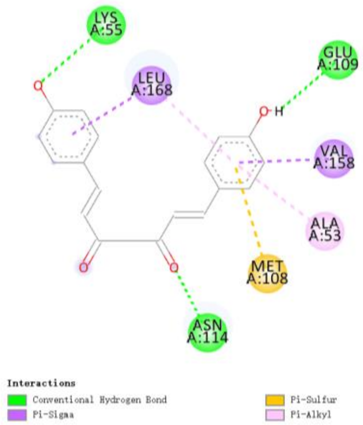
</td></tr>
<tr><td></td><td>1,2-Dihydrocurcumin</td><td>Hydrogen Bond: Lys55, Arg69, Glu73, Met111 (4)
Hydrophobic: Ile32, Ala53, Lys55, Val158</td><td>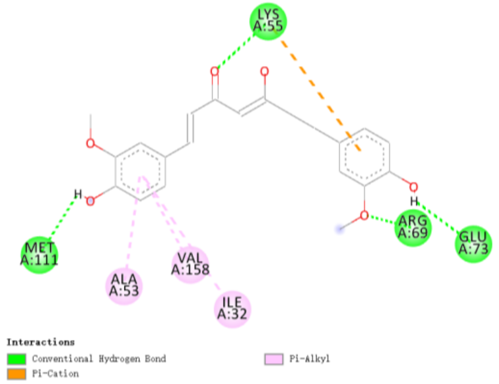
</td></tr>
</table>

	Turmeronol A	Hydrogen Bond: Lys55, Glu73 (2)<br>Hydrophobic: Val40, Ala53, Lys55, Glu73, Met108, Val158, Leu168	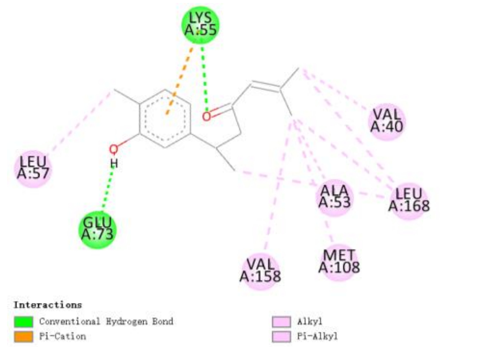
MAPK14	Bisdemethoxycurcumin	Hydrogen Bond: Glu71, Met109, Gly110, Asp112 (4)<br>Hydrophobic: Lys53, Ile84, Ala111, Leu167	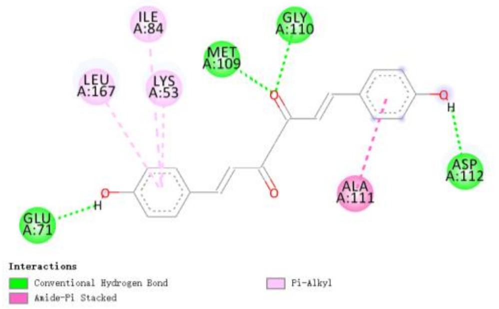
	Curcumenolactone A	Hydrogen Bond: Gly110 (1)<br>Hydrophobic: Val30, Val38, Ala51, Ile84, Ala157, Leu167	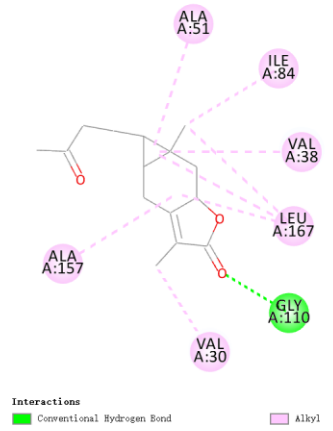

	Curcumenolactone B	Hydrogen Bond: Met109, Gly110 (2)<br>Hydrophobic: Val38, Ala51, Lys53, Ile84, Leu104, Ala157, Leu167	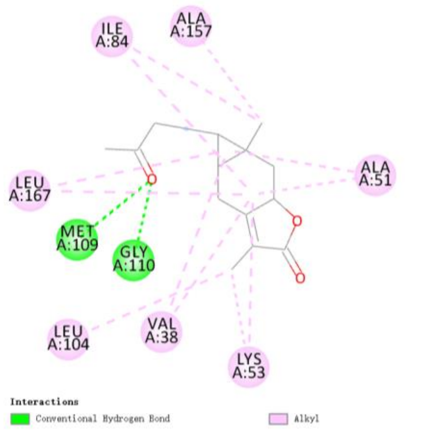
	1,2-Dihydrocurcumin	Hydrogen Bond: Val30, Met109, Gly110 (3)<br>Hydrophobic: Val30, Ala51, Lys53	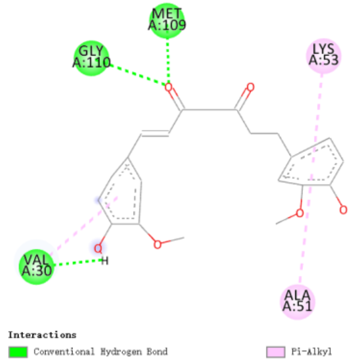
	Turmeronol A	Hydrogen Bond: Ala51, Met109 (2)<br>Hydrophobic: Val38, Ala51, Lys53, Leu75, Ile84, Leu104, Ala157	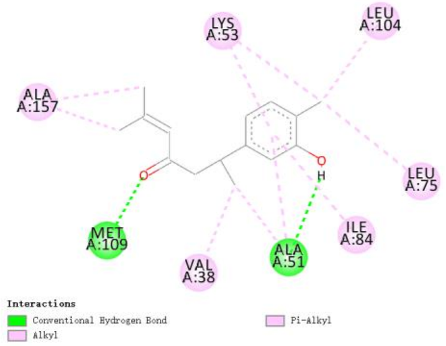

IL1B	Ar-turmerone	Hydrogen Bond: -<br>Hydrophobic: Leu26, Lys77, Leu80, Leu82	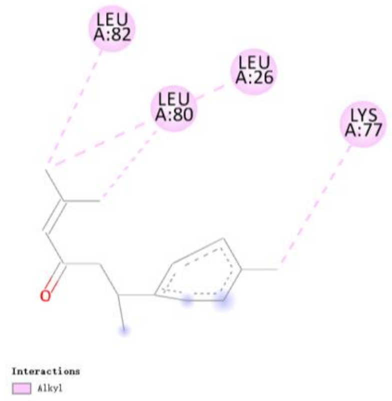
	Curcumalactone	Hydrogen Bond: Leu26 (1)<br>Hydrophobic: Leu80, Leu82, Pro131, Val132	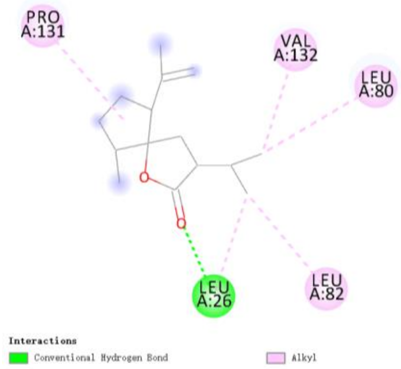
	Bisdemethoxycurcumin	Hydrogen Bond: Glu25, Leu26, Lys74, Gln81, Val132 (5)	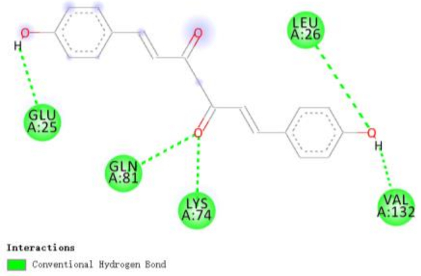

	Curcumenolactone A	Hydrogen Bond: Lys74, Gln81 (2)<br>Hydrophobic: Lys74	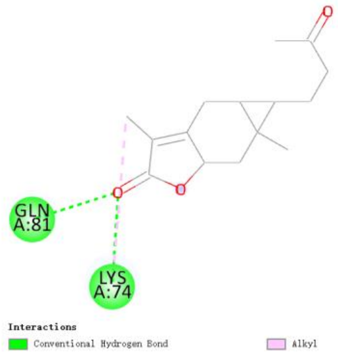
	1,2-Dihydrocurcumin	Hydrogen Bond: Glu25, Leu26, Lys74, Gln81, Val132 (6)	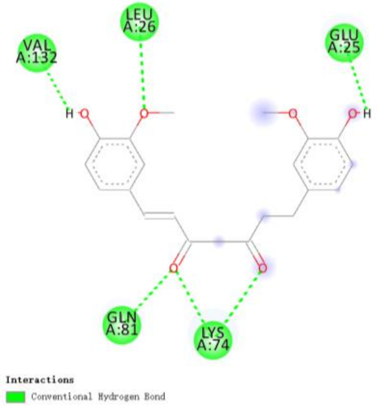
