# Supplementary material for: Network Pharmacology Prediction and Molecular Docking-Based Strategy to Discover the Potential Pharmacological Mechanism of Wen-Yu-Jin against Pulmonary Fibrosis in a Mouse Model
Source: Evid Based Complement Alternat Med. 2022 Feb 10;2022:7753508. doi: 10.1155/2022/7753508 (PMC8853792; doi:10.1155/2022/7753508)
Supplement: Supplementary Materials — Supplementary Table S1: information about WYJ-related targets. Supplementary Table S2: information about PF-related targets. Supplementary Table S3: targets information at the intersection of drug targets and disease targets. Supplementary Table S4: 48 key targets of WYJ against PF. Supplementary Table S5: core gene information filtered according to the “betweenness,” “closeness,” and “degree” values. Supplementary-Molecular Docking: information about molecular docking. [file 7753508.f1.zip › 7753508.f1/Supplementary Table S1.pdf]

Supplementary Table S1 WYJ-related targets

Ingredient of WYJ	Target
(Z)-p-Methoxycinnamic acid	CHRNB2
(Z)-p-Methoxycinnamic acid	CHRNA4
(Z)-p-Methoxycinnamic acid	ITGB2
(Z)-p-Methoxycinnamic acid	ICAM1
(Z)-p-Methoxycinnamic acid	ITGAL
(Z)-p-Methoxycinnamic acid	TAS1R3
(Z)-p-Methoxycinnamic acid	TAS1R1
(Z)-p-Methoxycinnamic acid	AKR1B1
(Z)-p-Methoxycinnamic acid	CA2
(Z)-p-Methoxycinnamic acid	CA7
(Z)-p-Methoxycinnamic acid	CA1
(Z)-p-Methoxycinnamic acid	CA12
(Z)-p-Methoxycinnamic acid	CA14
(Z)-p-Methoxycinnamic acid	CA9
(Z)-p-Methoxycinnamic acid	CA4
(Z)-p-Methoxycinnamic acid	SLC16A1
(Z)-p-Methoxycinnamic acid	CA5A
(Z)-p-Methoxycinnamic acid	CA3
(Z)-p-Methoxycinnamic acid	CA5B
(Z)-p-Methoxycinnamic acid	TLR4
(Z)-p-Methoxycinnamic acid	ESR2
(Z)-p-Methoxycinnamic acid	MMP1
(Z)-p-Methoxycinnamic acid	HCAR2
(Z)-p-Methoxycinnamic acid	CA6
(Z)-p-Methoxycinnamic acid	ALOX5
(Z)-p-Methoxycinnamic acid	MAOB
(Z)-p-Methoxycinnamic acid	F3
(Z)-p-Methoxycinnamic acid	PAM
(Z)-p-Methoxycinnamic acid	AKR1B10
(Z)-p-Methoxycinnamic acid	KDM4E
(Z)-p-Methoxycinnamic acid	KDM3A
(Z)-p-Methoxycinnamic acid	KDM2A
(Z)-p-Methoxycinnamic acid	KDM5C
(Z)-p-Methoxycinnamic acid	KDM4A
(Z)-p-Methoxycinnamic acid	KDM4C
(Z)-p-Methoxycinnamic acid	KMO
(Z)-p-Methoxycinnamic acid	RELA
(Z)-p-Methoxycinnamic acid	APEX1
(Z)-p-Methoxycinnamic acid	NOTUM
(Z)-p-Methoxycinnamic acid	MMP2
(Z)-p-Methoxycinnamic acid	CA13
(Z)-p-Methoxycinnamic acid	EGLN3
(Z)-p-Methoxycinnamic acid	PTGDR2
(Z)-p-Methoxycinnamic acid	PTPN1
(Z)-p-Methoxycinnamic acid	PTGS2
(Z)-p-Methoxycinnamic acid	MMP8
(Z)-p-Methoxycinnamic acid	SLC22A12
(Z)-p-Methoxycinnamic acid	FOLH1
(Z)-p-Methoxycinnamic acid	CPA3
(Z)-p-Methoxycinnamic acid	GRK2
(Z)-p-Methoxycinnamic acid	MAPK10
(Z)-p-Methoxycinnamic acid	MMP9
(Z)-p-Methoxycinnamic acid	APP
(Z)-p-Methoxycinnamic acid	KDM4B
(Z)-p-Methoxycinnamic acid	KDM5B

436 WYJ-related targets
CHRNB2
CHRNA4
ITGB2
ICAM1
ITGAL
TAS1R3
TAS1R1
AKR1B1
CA2
CA7
CA1
CA12
CA14
CA9
CA4
SLC16A1
CA5A
CA3
CA5B
TLR4
ESR2
MMP1
HCAR2
CA6
ALOX5
MAOB
F3
PAM
AKR1B10
KDM4E
KDM3A
KDM2A
KDM5C
KDM4A
KDM4C
KMO
RELA
APEX1
NOTUM
MMP2
CA13
EGLN3
PTGDR2
PTPN1
PTGS2
MMP8
SLC22A12
FOLH1
CPA3
GRK2
MAPK10
MMP9
APP
KDM4B
KDM5B

(Z)-p-Methoxycinnamic acid	MIF	MIF
(Z)-p-Methoxycinnamic acid	ACE	ACE
(Z)-p-Methoxycinnamic acid	SLC16A3	SLC16A3
(Z)-p-Methoxycinnamic acid	PTPRC	PTPRC
(Z)-p-Methoxycinnamic acid	EDNRB	EDNRB
(Z)-p-Methoxycinnamic acid	REN	REN
(Z)-p-Methoxycinnamic acid	PTGER4	PTGER4
(Z)-p-Methoxycinnamic acid	GSTA1	GSTA1
(Z)-p-Methoxycinnamic acid	GSTP1	GSTP1
(Z)-p-Methoxycinnamic acid	NQO2	NQO2
(Z)-p-Methoxycinnamic acid	GSK3B	GSK3B
(Z)-p-Methoxycinnamic acid	BACE1	BACE1
(Z)-p-Methoxycinnamic acid	CPB2	CPB2
(Z)-p-Methoxycinnamic acid	CPN1	CPN1
1,2-Dihydrocurcumin	ALOX5	HTR1A
1,2-Dihydrocurcumin	HTR1A	NFE2L2
1,2-Dihydrocurcumin	NFE2L2	EGFR
1,2-Dihydrocurcumin	EGFR	PTGS1
1,2-Dihydrocurcumin	CA2	ALOX15
1,2-Dihydrocurcumin	PTGS1	PRKCE
1,2-Dihydrocurcumin	CA7	EP300
1,2-Dihydrocurcumin	APP	HSD11B1
1,2-Dihydrocurcumin	CA1	NOS2
1,2-Dihydrocurcumin	ALOX15	PTGES
1,2-Dihydrocurcumin	CA6	TLR9
1,2-Dihydrocurcumin	MMP9	RAF1
1,2-Dihydrocurcumin	CA12	BRAF
1,2-Dihydrocurcumin	PRKCE	MET
1,2-Dihydrocurcumin	CA9	SYK
1,2-Dihydrocurcumin	CA4	GCGR
1,2-Dihydrocurcumin	EP300	WEE1
1,2-Dihydrocurcumin	CA13	CHEK2
1,2-Dihydrocurcumin	CA5B	AURKB
1,2-Dihydrocurcumin	F3	RPS6KB1
1,2-Dihydrocurcumin	HSD11B1	AURKA
1,2-Dihydrocurcumin	NOS2	ABCB1
1,2-Dihydrocurcumin	BACE1	ABCC1
1,2-Dihydrocurcumin	PTGES	PDK1
1,2-Dihydrocurcumin	TLR4	CHEK1
1,2-Dihydrocurcumin	TLR9	PARP1
1,2-Dihydrocurcumin	RAF1	PIK3CD
1,2-Dihydrocurcumin	BRAF	ALDH2
1,2-Dihydrocurcumin	MET	CDK1
1,2-Dihydrocurcumin	SYK	HSD17B2
1,2-Dihydrocurcumin	GCGR	DNM1
1,2-Dihydrocurcumin	GSK3B	CAMK2A
1,2-Dihydrocurcumin	WEE1	AKT2
1,2-Dihydrocurcumin	CHEK2	MELK
1,2-Dihydrocurcumin	AURKB	P2RY12
1,2-Dihydrocurcumin	RPS6KB1	AGTR1
1,2-Dihydrocurcumin	AURKA	ALOX5AP
1,2-Dihydrocurcumin	ABCB1	PIK3CA
1,2-Dihydrocurcumin	ABCC1	DRD3
1,2-Dihydrocurcumin	PDK1	HDAC8
1,2-Dihydrocurcumin	CHEK1	CCNE1
1,2-Dihydrocurcumin	PARP1	CDK2
1,2-Dihydrocurcumin	PIK3CD	CCNE2
1,2-Dihydrocurcumin	ALDH2	GABRB3

1,2-Dihydrocurcumin	CDK1	GABRA3
1,2-Dihydrocurcumin	HSD17B2	GABRG2
1,2-Dihydrocurcumin	DNM1	GABRA1
1,2-Dihydrocurcumin	CAMK2A	GABRB2
1,2-Dihydrocurcumin	AKT2	GABRA2
1,2-Dihydrocurcumin	MELK	GABRA6
1,2-Dihydrocurcumin	P2RY12	GABRA5
1,2-Dihydrocurcumin	AGTR1	PSEN2
1,2-Dihydrocurcumin	ALOX5AP	PSENEN
1,2-Dihydrocurcumin	PIK3CA	NCSTN
1,2-Dihydrocurcumin	DRD3	APH1A
1,2-Dihydrocurcumin	HDAC8	PSEN1
ar-turmerone	CCNE1	APH1B
ar-turmerone	CDK2	MGLL
ar-turmerone	CCNE2	DRD2
ar-turmerone	GABRB3	DRD4
ar-turmerone	GABRA3	SRD5A1
ar-turmerone	GABRG2	CHRM1
ar-turmerone	GABRA1	GRM1
ar-turmerone	GABRB2	HTR2C
ar-turmerone	GABRG2	PREP
ar-turmerone	GABRA2	P2RX7
ar-turmerone	GABRG2	CYP19A1
ar-turmerone	GABRB3	OPRL1
ar-turmerone	GABRB3	HCRTR2
ar-turmerone	GABRG2	CACNA1B
ar-turmerone	GABRA6	GRM5
ar-turmerone	GABRB3	EPHX2
ar-turmerone	GABRB3	MPO
ar-turmerone	GABRG2	C5AR1
ar-turmerone	GABRG2	AKR1C3
ar-turmerone	GABRA1	HCRTR1
ar-turmerone	GABRA5	MTNR1A
ar-turmerone	PSEN2	MTNR1B
ar-turmerone	PSENEN	KCNN4
ar-turmerone	NCSTN	KCNA3
ar-turmerone	APH1A	MC4R
ar-turmerone	PSEN1	EPHX1
ar-turmerone	APH1B	LTA4H
ar-turmerone	MGLL	TGFBR1
ar-turmerone	DRD2	FCGR1A
ar-turmerone	DRD4	CRHR1
ar-turmerone	SRD5A1	CHRM4
ar-turmerone	CHRM1	HTR2B
ar-turmerone	GRM1	ADRA2A
ar-turmerone	HTR2C	HTR1B
ar-turmerone	PREP	ADRA2C
ar-turmerone	P2RX7	HRH2
ar-turmerone	CYP19A1	ADRA2B
ar-turmerone	OPRL1	HTR1D
ar-turmerone	HCRTR2	CHRM5
ar-turmerone	CACNA1B	DRD1
ar-turmerone	GRM5	CHRM2
ar-turmerone	EPHX2	ADRA1D
ar-turmerone	MPO	ADRA1A
ar-turmerone	C5AR1	HRH1
ar-turmerone	AKR1C3	CHRM3
ar-turmerone	HCRTR1	HTR6

ar-turmerone	MTNR1A	TACR3
ar-turmerone	MTNR1B	PIK3CB
ar-turmerone	HSD17B2	TSPO
ar-turmerone	KCNN4	DNMT3A
ar-turmerone	KCNA3	SIRT2
ar-turmerone	MC4R	BRD4
ar-turmerone	EPHX1	PABPC1
ar-turmerone	LTA4H	PDE4B
ar-turmerone	PTGDR2	ADH1A
ar-turmerone	TGFBR1	TDO2
ar-turmerone	FCGR1A	STAT3
ar-turmerone	CRHR1	CDK5
ar-turmerone	CHRM4	CDK5R1
ar-turmerone	HTR2B	HDAC3
ar-turmerone	ADRA2A	NCOR2
ar-turmerone	HTR1B	CHUK
ar-turmerone	ADRA2C	IKBKB
ar-turmerone	HRH2	IKBKG
ar-turmerone	ADRA2B	MAOA
ar-turmerone	HTR1D	TOP2A
ar-turmerone	CHRM5	GLO1
ar-turmerone	DRD1	SRC
ar-turmerone	CHRM2	PLCG1
ar-turmerone	ADRA1D	EEF2K
ar-turmerone	ADRA1A	SERPINE1
ar-turmerone	HRH1	MTOR
ar-turmerone	DRD3	HSD17B1
ar-turmerone	CHRM3	MMP13
ar-turmerone	HTR6	CTSL
ar-turmerone	TACR3	ABL1
ar-turmerone	PIK3CB	PDGFRB
ar-turmerone	PIK3CA	FLT3
ar-turmerone	TSPO	TOP1
ar-turmerone	DNMT3A	HSP90AA1
ar-turmerone	SIRT2	ROCK2
ar-turmerone	BRD4	MCL1
ar-turmerone	PABPC1	GLI2
ar-turmerone	PDE4B	GLI1
ar-turmerone	ADH1A	PLK1
ar-turmerone	GABRA5	ESR1
ar-turmerone	TDO2	HDAC7
ar-turmerone	STAT3	CFD
bisdemethoxycurcumin	CDK5	ALPL
bisdemethoxycurcumin	CDK5R1	STAT6
bisdemethoxycurcumin	HDAC3	PIM1
bisdemethoxycurcumin	NCOR2	BMP1
bisdemethoxycurcumin	CHUK	CLK3
bisdemethoxycurcumin	IKBKB	ADH4
bisdemethoxycurcumin	IKBKG	ADH1B
bisdemethoxycurcumin	BACE1	ADH1C
bisdemethoxycurcumin	MAOA	TYR
bisdemethoxycurcumin	APP	CES1
bisdemethoxycurcumin	EP300	CES2
bisdemethoxycurcumin	PTGES	ALDH1A1
bisdemethoxycurcumin	TLR9	PIK3R1
bisdemethoxycurcumin	F3	F2
bisdemethoxycurcumin	HSD11B1	PRSS1
bisdemethoxycurcumin	TOP2A	CTRC

bisdemethoxycurcumin	GLO1	CYP17A1
bisdemethoxycurcumin	SRC	CTSK
bisdemethoxycurcumin	PLCG1	CTSB
bisdemethoxycurcumin	EEF2K	CTSH
bisdemethoxycurcumin	ALOX5	SLC6A4
bisdemethoxycurcumin	AURKA	POLB
bisdemethoxycurcumin	SERPINE1	TAAR1
bisdemethoxycurcumin	CA7	SLC6A3
bisdemethoxycurcumin	CA1	PLA2G6
bisdemethoxycurcumin	CA6	AR
bisdemethoxycurcumin	CA14	PGR
bisdemethoxycurcumin	CA9	KCNH2
bisdemethoxycurcumin	CA4	QPCT
bisdemethoxycurcumin	CA5B	FAAH
bisdemethoxycurcumin	CA5A	BCHE
bisdemethoxycurcumin	MTOR	LIPE
bisdemethoxycurcumin	HSD17B2	PDE10A
bisdemethoxycurcumin	HSD17B1	CCR4
bisdemethoxycurcumin	GSK3B	KCNMA1
bisdemethoxycurcumin	CDK1	CYP11B1
bisdemethoxycurcumin	EGFR	CYP11B2
bisdemethoxycurcumin	MMP13	NOS1
bisdemethoxycurcumin	MMP8	NOS3
bisdemethoxycurcumin	NFE2L2	NR1I2
bisdemethoxycurcumin	PTGS1	IDH1
bisdemethoxycurcumin	CTSL	HRH3
bisdemethoxycurcumin	DNM1	HRH4
bisdemethoxycurcumin	RAF1	TRPA1
bisdemethoxycurcumin	BRAF	FNTB
bisdemethoxycurcumin	ABL1	FNTA
bisdemethoxycurcumin	PDGFRB	ATP12A
bisdemethoxycurcumin	FLT3	PPARG
bisdemethoxycurcumin	TOP1	CDC25C
bisdemethoxycurcumin	AGTR1	CDC25A
bisdemethoxycurcumin	MET	CDC25B
bisdemethoxycurcumin	AURKB	PPARA
bisdemethoxycurcumin	RPS6KB1	PPARD
bisdemethoxycurcumin	PDK1	NR3C2
bisdemethoxycurcumin	HSP90AA1	TAS2R31
bisdemethoxycurcumin	ROCK2	RPS6KA5
bisdemethoxycurcumin	MCL1	PRKCA
bisdemethoxycurcumin	GLI2	HDAC6
bisdemethoxycurcumin	GLI1	HTR2A
bisdemethoxycurcumin	PLK1	CTSS
bisdemethoxycurcumin	CHEK1	TTL
bisdemethoxycurcumin	WEE1	HMGCR
bisdemethoxycurcumin	ESR1	GRK6
bisdemethoxycurcumin	CHEK2	HSD17B3
bisdemethoxycurcumin	HDAC7	PSMB5
bisdemethoxycurcumin	CFD	PPP2CA
bisdemethoxycurcumin	ABCC1	PARP2
bisdemethoxycurcumin	AKR1B1	SHBG
bisdemethoxycurcumin	ALPL	CAPN1
bisdemethoxycurcumin	STAT6	HSD11B2
bisdemethoxycurcumin	PIM1	HMOX1
bisdemethoxycurcumin	BMP1	GPR139
bisdemethoxycurcumin	CLK3	F2R
cuminal	ADH4	PARP3

cuminal	ADH1B	PARP4
cuminal	ADH1C	TNKS
cuminal	TYR	NR1I3
cuminal	CES1	MAP2K1
cuminal	CES2	DPP4
cuminal	ALDH1A1	RIPK1
cuminal	GSK3B	IL1B
cuminal	SRD5A1	CDC7
Curcumalactone	GABRA2	MAPKAPK2
Curcumalactone	GABRB2	MMP3
Curcumalactone	GABRG2	GSK3A
Curcumalactone	PIK3CD	PDCD4
Curcumalactone	PIK3R1	ECE1
Curcumalactone	F2	MAPK14
Curcumalactone	PRSS1	CYP1A2
Curcumalactone	PTGS2	MME
Curcumalactone	CTRC	PDE7A
Curcumalactone	HSD11B1	FAP
Curcumalactone	CYP19A1	PTAFR
Curcumalactone	CYP17A1	SCN2A
Curcumalactone	PREP	SCN10A
Curcumalactone	CTSK	RPS6KA3
Curcumalactone	CTSL	IARS
Curcumalactone	CTSB	PTPN11
Curcumalactone	CTSH	JAK2
Curcumalactone	PTPN1	ADAM17
Curcumalactone	EPHX1	CSF1R
Curcumalactone	ADRA2A	DHODH
Curcumalactone	ADRA2C	SIRT1
Curcumalactone	ADRA2B	CDK4
Curcumalactone	SLC6A4	DBF4
Curcumalactone	POLB	CCND1
Curcumalactone	TAAR1	GABRA4
Curcumalactone	HTR1A	PLA2G1B
Curcumalactone	SLC6A3	CCR5
Curcumalactone	PLA2G6	ABCG2
Curcumalactone	ALDH2	SRD5A2
Curcumalactone	ADRA1A	TACR1
Curcumalactone	AR	CMA1
Curcumalactone	PGR	CTSG
Curcumalactone	KCNH2	IGF1R
Curcumalactone	NOS2	HLCS
Curcumalactone	QPCT	LRRK2
Curcumalactone	FAAH	DYRK1A
Curcumalactone	BCHE	NLRP3
Curcumalactone	LIPE	MDM2
Curcumalactone	ABCC1	CTSC
Curcumalactone	TOP2A	CTSF
Curcumalactone	PDE10A	CTSV
Curcumalactone	GRM5	KCNA5
Curcumalactone	PTGES	CTSD
Curcumalactone	CCR4	TNKS2
Curcumalactone	KCNMA1	CHRNB4
Curcumalactone	CYP11B1	CHRNA3
Curcumalactone	CYP11B2	PRKCG
Curcumalactone	P2RX7	PRKCD
Curcumalactone	NOS1	JAK1
Curcumalactone	NOS3	PER2

Curcumalactone	NR1I2	THRB
Curcumalactone	IDH1	EDNRA
Curcumalactone	HRH3	PRKCB
Curcumalactone	HRH4	PRKCQ
Curcumalactone	CHRNA4	CYP2C9
Curcumalactone	GSK3B	JAK3
Curcumalactone	TRPA1	TYK2
Curcumalactone	TGFBR1	SLC6A2
Curcumenolactone A	FNTB	RASGRP1
Curcumenolactone A	FNTA	IL6ST
Curcumenolactone A	GABRA1	MAPK8
Curcumenolactone A	GABRB2	CEL
Curcumenolactone A	GABRG2	KDR
Curcumenolactone A	PSEN2	KIF11
Curcumenolactone A	PSENEN	ABCC9
Curcumenolactone A	NCSTN	KCNJ11
Curcumenolactone A	APH1A	NR3C1
Curcumenolactone A	PSEN1	AOC3
Curcumenolactone A	APH1B	BRD2
Curcumenolactone A	CYP19A1	SERPINA6
Curcumenolactone A	AR	FABP1
Curcumenolactone A	PTPN1	FKBP1A
Curcumenolactone A	PGR	GPBAR1
Curcumenolactone A	ATP12A	GLUL
Curcumenolactone A	PPARG	KAT2B
Curcumenolactone A	CDC25C	IDO1
Curcumenolactone A	CDC25A	IL6
Curcumenolactone A	CDC25B	LIPA
Curcumenolactone A	HSD11B1	MPEG1
Curcumenolactone A	PPARA	NPC1L1
Curcumenolactone A	PPARD	RORA
Curcumenolactone A	NR3C2	FDFT1
Curcumenolactone A	ESR1	TRPM8
Curcumenolactone A	ESR2	ATP4A
Curcumenolactone A	TAS2R31	ATP4B
Curcumenolactone A	P2RX7	CCNB1
Curcumenolactone A	RPS6KA5	CCNA2
Curcumenolactone A	MTNR1A	CCNA1
Curcumenolactone A	MTNR1B	DRD5
Curcumenolactone A	CYP17A1	AKT1
Curcumenolactone A	CTSK	PRKCZ
Curcumenolactone A	NQO2	FGFR1
Curcumenolactone A	PRKCA	ECE2
Curcumenolactone A	HDAC6	HTR3A
Curcumenolactone A	PARP1	MYLK
Curcumenolactone A	HSD17B2	CASR
Curcumenolactone A	HTR2A	EGLN1
Curcumenolactone A	CTSS	RET
Curcumenolactone A	CTSB	RIPK2
Curcumenolactone A	TTL	P2RX3
Curcumenolactone A	BRD4	PDE2A
Curcumenolactone A	PTGS2	PIM3
Curcumenolactone A	HMGCR	CSNK1G1
Curcumenolactone A	MAOB	CSNK1A1
Curcumenolactone A	GRK6	CSNK1D
Curcumenolactone A	PTGS1	FPR1
Curcumenolactone A	CACNA1B	CLK2
Curcumenolactone A	HSD17B3	CALM1

Curcumenolactone A	PSMB5
Curcumenolactone A	PPP2CA
Curcumenolactone A	MPO
Curcumenolactone A	PARP2
Curcumenolactone A	SHBG
Curcumenolactone A	CAPN1
Curcumenolactone A	EPHX1
Curcumenolactone A	HSD11B2
Curcumenolactone A	HTR2C
Curcumenolactone A	F2
Curcumenolactone A	PRSS1
Curcumenolactone A	HMOX1
Curcumenolactone A	GPR139
Curcumenolactone A	CTSL
Curcumenolactone A	F2R
Curcumenolactone A	PARP3
Curcumenolactone A	PARP4
Curcumenolactone A	TNKS
Curcumenolactone A	NR1I3
Curcumenolactone A	MAOA
Curcumenolactone A	MAP2K1
Curcumenolactone A	DPP4
Curcumenolactone A	RIPK1
Curcumenolactone A	IL1B
Curcumenolactone A	CA2
Curcumenolactone A	CDC7
Curcumenolactone A	MAPKAPK2
Curcumenolactone A	MMP3
Curcumenolactone A	GSK3A
Curcumenolactone A	MMP9
Curcumenolactone A	MMP1
Curcumenolactone A	PDCD4
Curcumenolactone A	GABRA1
Curcumenolactone A	ECE1
Curcumenolactone A	GABRA5
Curcumenolactone A	MAPK14
Curcumenolactone A	CYP1A2
Curcumenolactone A	TSPO
Curcumenolactone A	MME
Curcumenolactone A	PDE7A
Curcumenolactone A	FAP
Curcumenolactone A	HRH1
Curcumenolactone A	PTAFR
Curcumenolactone A	SCN2A
Curcumenolactone A	SCN10A
Curcumenolactone A	RPS6KA3
Curcumenolactone A	HTR2B
Curcumenolactone A	HDAC8
Curcumenolactone A	CYP11B1
Curcumenolactone A	IARS
Curcumenolactone A	PTPN11
Curcumenolactone A	CYP11B2
Curcumenolactone A	JAK2
Curcumenolactone A	ADAM17
Curcumenolactone A	CSF1R
Curcumenolactone A	DHODH
Curcumenolactone A	KCNN4
Curcumenolactone A	SIRT1

TERT
MAPK1
NR1D1
DHFR
NPY5R
PLA2G2A
PHLPP2
PDPK1
MPI
BACE2
PDE5A
MAPK9
CNR1
NAMPT
TRPC6
SIGMAR1
FABP4
FABP3
FABP5
CD81
NR1H4
CYP51A1
NR1H3
PTPRF
PTPN2
ACP1
G6PD
SCD
CREBBP
ADRB2
ADRB1
ADRB3
PRKDC

Curcumenolactone B	CDC7
Curcumenolactone B	CDK4
Curcumenolactone B	DBF4
Curcumenolactone B	CCND1
Curcumenolactone B	FNTB
Curcumenolactone B	FNTA
Curcumenolactone B	GABRB3
Curcumenolactone B	GABRA4
Curcumenolactone B	GABRG2
Curcumenolactone B	GABRB3
Curcumenolactone B	GABRG2
Curcumenolactone B	GABRA5
Curcumenolactone B	PSEN2
Curcumenolactone B	PSENEN
Curcumenolactone B	NCSTN
Curcumenolactone B	APH1A
Curcumenolactone B	PSEN1
Curcumenolactone B	APH1B
Curcumenolactone B	CYP19A1
Curcumenolactone B	AR
Curcumenolactone B	PGR
Curcumenolactone B	PTPN1
Curcumenolactone B	ATP12A
Curcumenolactone B	PPARG
Curcumenolactone B	CDC25B
Curcumenolactone B	CDC25C
Curcumenolactone B	NR3C2
Curcumenolactone B	CDC25A
Curcumenolactone B	PPARA
Curcumenolactone B	PPARD
Curcumenolactone B	HSD11B1
Curcumenolactone B	ESR1
Curcumenolactone B	ESR2
Curcumenolactone B	TAS2R31
Curcumenolactone B	CYP17A1
Curcumenolactone B	P2RX7
Curcumenolactone B	MTNR1A
Curcumenolactone B	MTNR1B
Curcumenolactone B	MAOB
Curcumenolactone B	RPS6KA5
Curcumenolactone B	NQO2
Curcumenolactone B	PTGS1
Curcumenolactone B	HSD17B2
Curcumenolactone B	PRKCA
Curcumenolactone B	HDAC6
Curcumenolactone B	PLA2G1B
Curcumenolactone B	MAOA
Curcumenolactone B	PTGS2
Curcumenolactone B	TTL
Curcumenolactone B	CTSK
Curcumenolactone B	HMGCR
Curcumenolactone B	CCR5
Curcumenolactone B	PARP1
Curcumenolactone B	HSD17B3
Curcumenolactone B	CDC7
Curcumenolactone B	GPR139
Curcumenolactone B	CACNA1B
Curcumenolactone B	PPP2CA

Curcumenolactone B	SHBG
Curcumenolactone B	CYP1A2
Curcumenolactone B	SYK
Curcumenolactone B	HTR2C
Curcumenolactone B	MMP3
Curcumenolactone B	MMP9
Curcumenolactone B	MMP1
Curcumenolactone B	PARP3
Curcumenolactone B	PARP2
Curcumenolactone B	PARP4
Curcumenolactone B	TNKS
Curcumenolactone B	CES2
Curcumenolactone B	HTR2A
Curcumenolactone B	ABCG2
Curcumenolactone B	BRD4
Curcumenolactone B	CAPN1
Curcumenolactone B	SRD5A2
Curcumenolactone B	CTSS
Curcumenolactone B	CTSL
Curcumenolactone B	CTSB
Curcumenolactone B	MPO
Curcumenolactone B	NR1I3
Curcumenolactone B	F2
Curcumenolactone B	PRSS1
Curcumenolactone B	CA2
Curcumenolactone B	IL1B
Curcumenolactone B	CYP11B2
Curcumenolactone B	TACR1
Curcumenolactone B	PDCD4
Curcumenolactone B	CMA1
Curcumenolactone B	CTSG
Curcumenolactone B	TSPO
Curcumenolactone B	GABRA1
Curcumenolactone B	HDAC8
Curcumenolactone B	FAP
Curcumenolactone B	GABRA5
Curcumenolactone B	AKR1B1
Curcumenolactone B	DPP4
Curcumenolactone B	SCN2A
Curcumenolactone B	SCN10A
Curcumenolactone B	MAPK14
Curcumenolactone B	MAPKAPK2
Curcumenolactone B	RPS6KA3
Curcumenolactone B	HMOX1
Curcumenolactone B	GRK6
Curcumenolactone B	IARS
Curcumenolactone B	PTPN11
Curcumenolactone B	IGF1R
Curcumenolactone B	EPHX1
Curcumenolactone B	HLCS
Curcumenolactone B	LRRK2
Curcumenolactone B	GSK3A
Curcumenolactone B	ADAM17
Curcumenolactone B	DYRK1A
Epicurzerenone	SLC6A4
Epicurzerenone	SLC6A3
Epicurzerenone	PTGS2
Epicurzerenone	PABPC1

Epicurzerenone	PREP
Epicurzerenone	CTSK
Epicurzerenone	CTSL
Epicurzerenone	CTSB
Epicurzerenone	HSD17B2
Epicurzerenone	EPHX1
Epicurzerenone	NLRP3
Epicurzerenone	MDM2
Epicurzerenone	HCRTR1
Epicurzerenone	PTGS1
Epicurzerenone	NOS2
Epicurzerenone	AR
Epicurzerenone	HTR6
Epicurzerenone	CYP17A1
Epicurzerenone	CTSC
Epicurzerenone	CTSF
Epicurzerenone	CTSS
Epicurzerenone	CTSV
Epicurzerenone	CDC25B
Furanogermenone	SLC6A4
Furanogermenone	SLC6A3
Furanogermenone	AR
Furanogermenone	MTNR1A
Furanogermenone	PABPC1
Furanogermenone	CHRM2
Furanogermenone	CHRM1
Furanogermenone	KCNA5
Furanogermenone	CTSD
Furanogermenone	CTSS
Furanogermenone	CTSL
Furanogermenone	CTSB
Furanogermenone	PDE10A
Furanogermenone	TNKS2
germacrone-13-al	CHRNB4
germacrone-13-al	CHRNA3
germacrone-13-al	GABRB3
germacrone-13-al	GABRG2
germacrone-13-al	GABRA5
germacrone-13-al	PSEN2
germacrone-13-al	PSENEN
germacrone-13-al	NCSTN
germacrone-13-al	APH1A
germacrone-13-al	PSEN1
germacrone-13-al	APH1B
germacrone-13-al	PRKCG
germacrone-13-al	PRKCD
germacrone-13-al	EGFR
germacrone-13-al	JAK1
germacrone-13-al	JAK2
germacrone-13-al	PER2
germacrone-13-al	EPHX1
germacrone-13-al	THRB
germacrone-13-al	TGFBR1
germacrone-13-al	EDNRA
germacrone-13-al	GCGR
germacrone-13-al	PRKCB
germacrone-13-al	PRKCE
germacrone-13-al	PRKCQ

germacrone-13-al	CHRM4
germacrone-13-al	CHRM5
germacrone-13-al	CHRM2
germacrone-13-al	CHRM1
germacrone-13-al	CHRM3
germacrone-13-al	CYP2C9
germacrone-13-al	JAK3
germacrone-13-al	TYK2
germacrone-13-al	SLC6A2
germacrone-13-al	RASGRP1
germacrone-13-al	HSD17B2
germacrone-13-al	IL6ST
germacrone-13-al	KCNH2
germacrone-13-al	MAPK8
Gweicurculactone	CA3
Gweicurculactone	CA12
Gweicurculactone	CA14
Gweicurculactone	CA9
Gweicurculactone	CA13
Gweicurculactone	CA5B
Gweicurculactone	CA5A
Gweicurculactone	CEL
Gweicurculactone	KDR
Gweicurculactone	JAK1
Gweicurculactone	JAK2
Gweicurculactone	PRKCA
Gweicurculactone	AURKA
Gweicurculactone	PDE7A
Gweicurculactone	KIF11
procurcumadiol	ABCC9
procurcumadiol	KCNJ11
procurcumadiol	CDK5
procurcumadiol	CDK5R1
procurcumadiol	CYP19A1
procurcumadiol	AR
procurcumadiol	NR3C1
procurcumadiol	HSD11B1
procurcumadiol	ADAM17
procurcumadiol	AOC3
procurcumadiol	APP
procurcumadiol	BRD2
procurcumadiol	BRD4
procurcumadiol	CA3
procurcumadiol	CA4
procurcumadiol	CA5B
procurcumadiol	CA6
procurcumadiol	CA7
procurcumadiol	CA12
procurcumadiol	CA13
procurcumadiol	CA14
procurcumadiol	CES2
procurcumadiol	CDC7
procurcumadiol	SERPINA6
procurcumadiol	PTGS1
procurcumadiol	CYP11B1
procurcumadiol	CYP11B2
procurcumadiol	CYP17A1
procurcumadiol	ESR2

procurcumadiol	FABP1
procurcumadiol	FKBP1A
procurcumadiol	GPBAR1
procurcumadiol	GLUL
procurcumadiol	HMOX1
procurcumadiol	KAT2B
procurcumadiol	IDO1
procurcumadiol	IL6
procurcumadiol	LRRK2
procurcumadiol	LIPA
procurcumadiol	MPEG1
procurcumadiol	NR3C2
procurcumadiol	MGLL
procurcumadiol	CHRM1
procurcumadiol	MPO
procurcumadiol	NPC1L1
procurcumadiol	RORA
procurcumadiol	PPARD
procurcumadiol	PDE10A
procurcumadiol	PTAFR
procurcumadiol	PARP1
procurcumadiol	PGR
procurcumadiol	PTPN1
procurcumadiol	FDFT1
procurcumadiol	ABCC9
procurcumadiol	TAAR1
procurcumadiol	TRPM8
procurcumadiol	JAK1
procurcumadiol	JAK2
procurcumadiol	JAK3
procurcumadiol	TYK2
turmeronol A	KCNJ11
turmeronol A	ABCC9
turmeronol A	ATP4A
turmeronol A	ATP4B
turmeronol A	CCNB1
turmeronol A	CCNA2
turmeronol A	CCNA1
turmeronol A	CDK2
turmeronol A	CDK1
turmeronol A	CCNE1
turmeronol A	CDK4
turmeronol A	CDK2
turmeronol A	CCND1
turmeronol A	CDK5R1
turmeronol A	CDK5
turmeronol A	CDK5R1
turmeronol A	CHRNB4
turmeronol A	CHRNA3
turmeronol A	GABRB3
turmeronol A	GABRA3
turmeronol A	GABRG2
turmeronol A	GABRA2
turmeronol A	GABRB3
turmeronol A	GABRG2
turmeronol A	GABRB3
turmeronol A	GABRG2
turmeronol A	GABRA1

turmeronol A	GABRB3
turmeronol A	GABRG2
turmeronol A	GABRA5
turmeronol A	PIK3CD
turmeronol A	PIK3R1
turmeronol A	PSEN2
turmeronol A	PSENEN
turmeronol A	NCSTN
turmeronol A	APH1A
turmeronol A	PSEN1
turmeronol A	APH1B
turmeronol A	ALOX5
turmeronol A	NQO2
turmeronol A	DRD5
turmeronol A	MTNR1B
turmeronol A	HSD17B2
turmeronol A	AKT1
turmeronol A	PRKCZ
turmeronol A	NOS2
turmeronol A	KDR
turmeronol A	POLB
turmeronol A	MMP9
turmeronol A	MMP1
turmeronol A	MMP2
turmeronol A	FGFR1
turmeronol A	ECE2
turmeronol A	CA12
turmeronol A	CA9
turmeronol A	HTR1A
turmeronol A	HTR3A
turmeronol A	MYLK
turmeronol A	MC4R
turmeronol A	LRRK2
turmeronol A	CASR
turmeronol A	SRC
turmeronol A	EGLN1
turmeronol A	MAPK14
turmeronol A	TGFBR1
turmeronol A	GABRA1
turmeronol A	RET
turmeronol A	RIPK2
turmeronol A	TSPO
turmeronol A	P2RX3
turmeronol A	AKR1B1
turmeronol A	JAK3
turmeronol A	JAK1
turmeronol A	JAK2
turmeronol A	TYK2
turmeronol A	EPHX2
turmeronol A	IGF1R
turmeronol A	PDE2A
turmeronol A	PDE10A
turmeronol A	PIM1
turmeronol A	PARP1
turmeronol A	PIM3
turmeronol A	CSNK1G1
turmeronol A	CSNK1A1
turmeronol A	CSNK1D

turmeronol A	FPR1
turmeronol A	CDK5
turmeronol A	CLK2
turmeronol A	PTGS2
turmeronol A	CTSD
turmeronol A	CALM1
turmeronol A	CDK2
turmeronol A	CDK4
turmeronol A	CACNA1B
turmeronol A	FLT3
turmeronol A	GSK3B
turmeronol A	TERT
turmeronol A	MAPK1
turmeronol A	LTA4H
turmeronol A	HCRTR2
turmeronol A	HCRTR1
turmeronol A	NR1D1
turmeronol A	DHFR
turmeronol A	SIRT2
turmeronol A	NPY5R
turmeronol A	HSD17B1
turmeronol A	MMP13
turmeronol A	PLA2G2A
turmeronol A	PHLPP2
turmeronol A	MAPK8
turmeronol A	PDPK1
turmeronol A	MAPK10
turmeronol A	MPI
turmeronol A	CTSS
turmeronol A	BACE2
turmeronol A	KCNMA1
turmeronol A	PDE5A
turmeronol A	CTSV
turmeronol A	MAPK9
turmeronol A	CDC7
turmeronol A	GSK3A
turmeronol A	BRAF
turmeronol A	CNR1
turmeronol A	NAMPT
turmeronol A	TRPC6
turmeronol A	NR3C1
turmeronol A	CYP19A1
turmeronol A	PTGS2
turmeronol A	AR
turmeronol A	ATP12A
turmeronol A	HSD11B1
turmeronol A	FKBP1A
turmeronol A	NR3C2
turmeronol A	PTAFR
turmeronol A	LRRK2
turmeronol A	HSD11B2
turmeronol A	BRD4
turmeronol A	BRD2
turmeronol A	PGR
turmeronol A	CDC7
turmeronol A	SIGMAR1
turmeronol A	TAAR1
turmeronol A	APP

turmeronol A	CYP11B1
turmeronol A	CYP11B2
turmeronol A	CA2
turmeronol A	CA7
turmeronol A	CA1
turmeronol A	CA3
turmeronol A	CA6
turmeronol A	CA12
turmeronol A	CA14
turmeronol A	CA9
turmeronol A	CA4
turmeronol A	CA13
turmeronol A	CA5B
turmeronol A	NPC1L1
turmeronol A	IL6
turmeronol A	GLUL
turmeronol A	GPBAR1
turmeronol A	FABP1
turmeronol A	JAK3
turmeronol A	JAK1
turmeronol A	JAK2
turmeronol A	TYK2
turmeronol A	ABCC9
turmeronol A	FABP4
turmeronol A	PPARA
turmeronol A	FABP3
turmeronol A	FABP5
turmeronol A	PTPN1
turmeronol A	CD81
turmeronol A	PTPN11
turmeronol A	AKR1B10
turmeronol A	HMOX1
turmeronol A	NR1H4
turmeronol A	FAAH
turmeronol A	CYP51A1
turmeronol A	LIPA
turmeronol A	NR1H3
turmeronol A	PTPRF
turmeronol A	PTPN2
turmeronol A	PLA2G1B
turmeronol A	ACP1
turmeronol A	G6PD
turmeronol A	CYP17A1
turmeronol A	PDE10A
turmeronol A	SCD
turmeronol A	AOC3
turmeronol A	KCNA5
turmeronol A	SERPINA6
turmeronol A	SHBG
zedoarondiol	CDK5
zedoarondiol	CDK5R1
zedoarondiol	PTGS2
zedoarondiol	CYP19A1
zedoarondiol	HSD11B1
zedoarondiol	NR3C1
zedoarondiol	BRD4
zedoarondiol	BRD2
zedoarondiol	NR3C2

zedoarondiol	ATP12A
zedoarondiol	AR
zedoarondiol	CDC7
zedoarondiol	SERPINA6
zedoarondiol	SHBG
zedoarondiol	CA2
zedoarondiol	CA1
zedoarondiol	BACE1
zedoarondiol	CA7
zedoarondiol	CA3
zedoarondiol	CA6
zedoarondiol	CA12
zedoarondiol	CA14
zedoarondiol	CA9
zedoarondiol	CA4
zedoarondiol	CA13
zedoarondiol	CA5B
zedoarondiol	HSD11B2
zedoarondiol	JAK1
zedoarondiol	FKBP1A
zedoarondiol	JAK3
zedoarondiol	TYK2
zedoarondiol	SIGMAR1
zedoarondiol	LRRK2
zedoarondiol	JAK2
zedoarondiol	NPC1L1
zedoarondiol	NOS2
zedoarondiol	PGR
zedoarondiol	CREBBP
zedoarondiol	FABP4
zedoarondiol	FABP3
zedoarondiol	FABP5
zedoarondiol	CYP11B1
zedoarondiol	CYP11B2
zedoarondiol	MTNR1A
zedoarondiol	ABCC9
zedoarondiol	NOS1
zedoarondiol	NOS3
zedoarondiol	PTPN1
zedoarondiol	CD81
zedoarondiol	PTPN11
zedoarondiol	AKR1B10
zedoarondiol	MIF
zedoarondiol	APP
zedoarondiol	NR1H4
zedoarondiol	ADRB2
zedoarondiol	ADRB1
zedoarondiol	ADRB3
zedoarondiol	PTAFR
zedoarondiol	PRKDC
zedoarondiol	PDE10A
zedoarondiol	TRPM8
zedoarondiol	HMGCR
zedoarondiol	CYP17A1
zedoarondiol	PARP1
zedoarondiol	PIK3CD
zedoarondiol	PIK3CB
zedoarondiol	MAOA

zedoarondiol	NR1H3
zedoarondiol	PTPN2
zedoarondiol	PLA2G1B
zedoarondiol	ACP1
zedoarondiol	G6PD
zedoarondiol	AOC3
zedoarondiol	PTGES
zedoarondiol	SCD
zedoarondiol	MPO
zedoarondiol	CHEK1
zedoarondiol	TAAR1
