# Supplementary material for: Network Pharmacology Prediction and Molecular Docking-Based Strategy to Discover the Potential Pharmacological Mechanism of Wen-Yu-Jin against Pulmonary Fibrosis in a Mouse Model
Source: Evid Based Complement Alternat Med. 2022 Feb 10;2022:7753508. doi: 10.1155/2022/7753508 (PMC8853792; doi:10.1155/2022/7753508)
Supplement: Supplementary Materials — Supplementary Table S1: information about WYJ-related targets. Supplementary Table S2: information about PF-related targets. Supplementary Table S3: targets information at the intersection of drug targets and disease targets. Supplementary Table S4: 48 key targets of WYJ against PF. Supplementary Table S5: core gene information filtered according to the “betweenness,” “closeness,” and “degree” values. Supplementary-Molecular Docking: information about molecular docking. [file 7753508.f1.zip › 7753508.f1/Supplementary Table S2.pdf]

Supplementary Table S2 846 Targets associated with IPF

TERT
SFTPC
SFTPA2
MUC5B
ABCA3
TERC
LOC110806263
RTEL1
SFTPA1
RTEL1-TNFRSF6B
IL6
PARN
DSP
TNF
COPA
SFTPB
FAM13A
DPP9
ATP11A
STN1
IFNG
SFTPD
CXCL8
IL10
TGFB1
CRP
MUC1
HLA-DRB1
PDGFRB
IL4
IL1B
PDGFRA
ALB
CCL2
MMP1
PDGFB
FARSB
IL13
IL17A
ITGA3
ACE
ELANE
TLR4
STAT3
CCL5
MPO
MMP9
CFTR
SERPINE1
ICAM1
ACTC1
IL18
IL1RN
CXCR4
CD4
VEGFA

CD40LG
IL5
FOXP3
FLNA
CSF2
IL2RA
CXCR3
ADA
CCL3
CXCL10
CD8A
TIMP1
SLC2A1
CAV1
IL1A
HLA-DQB1
EDN1
CASR
TLR2
SCN5A
CHD7
FGF2
HMGB1
IL2
CSF3
CCL18
TNNI3
PTPRC
INPP5E
CCN2
TLR5
ELN
CXCL5
EGFR
AGT
FGF8
MIF
TINF2
CD34
CCL17
IGF1
ITGAM
SERPINA1
CCR6
SERPINH1
PRTN3
SCGB1A1
CCR4
CXCL9
CXCL1
FGFR1
LACTB
TP53
HMOX1
TNNT2
FBN1
CTLA4
MMP2

IFIH1
CCR7
WT1
HLA-DQA1
NKX2-1
MYH7
CXCL12
PPARG
UMOD
MBL2
FASLG
LRBA
IL1R1
CCL22
HLA-B
IL33
MIR21
TLR9
PIK3C2A
CHAT
TRIM21
REN
ELMOD2
IL2RB
SPP1
FN1
TTN
VCAM1
MMP12
BTK
CXCR2
AGTR1
MMP7
DMD
KIT
MMP8
NKX2-5
MUC5AC
STAT5B
EGF
THBD
APOE
SHH
B2M
TNFRSF13B
SPPL2C
RAG1
DES
MYD88
ACE2
COL1A1
AKT1
NEU1
GBA
FOXF1
NOS3
CTNNB1
CD79A

F2
BDNF
LMNA
HIF1A
BMP6
GPT
CR1
NPHS1
TGFB3
ITGB2
CDH1
JUN
CCR2
TOLLIP
AGER
HLA-A
CCL4
FLNC
H2AC18
TGFB2
IL13RA2
STAT4
CCL11
HGF
HARS1
THBS1
INS
BMP4
MYH6
COL3A1
CMA1
SELL
CHI3L1
CCR5
GAPDH
HPS1
TF
DKC1
SMPD1
POSTN
FCGR2A
FAS
CALCA
PKD1
C3
CXCR1
MAPK3
MYLK
MIR140
PDCD1
SPARC
LGALS3
COL1A2
IGFBP3
STING1
NPHP4
S100A1
ICOSLG

ATP4A
ALOX5
MAPT
ACP5
MRC1
GREM1
COL4A5
ENG
VIM
LEP
RNASE3
ATP12A
NLRP3
VCL
CYBB
SMAD3
F3
LTA
TLR3
MIR155
TNC
ADRB2
PDGFA
GSN
SMAD2
ICOS
ADM
HSPD1
MMP3
NOX4
IL9
NFKB1
NHP2
NOP10
TNFRSF11A
SERPINA3
IFNA1
TNFRSF1A
SMAD4
FGF7
SLPI
LAMP2
KRT18
FGFR3
GATA4
PRL
IL7R
ACTB
NOS1
CFH
HP
SIGLEC5
INVS
LOXL2
PMPCA
RYR1
MIR214
LIG4

NOD2
THPO
CACNA1C
FHIT
ARG1
FKTN
PTPN11
GRP
TAC1
MIR199A1
ENPP2
PKD2
SNCA
ADCY10
MIR30A
F5
CASP3
STAT6
TBX4
TGFBR1
PTEN
S100A4
AGTR2
LOX
CD274
NGF
DEFB4A
NPPB
AR
NCF2
DSG2
TERF1
VTN
NCF1
NPC2
CCN4
LAMA2
CYP1A1
TRPV1
TSLP
IGHE
IL15
PRKCD
WRAP53
LPAR1
THY1
CCND1
SMAD7
LAMC2
DDR1
CXCL2
TREM1
MIRLET7D
CD36
S100A12
ITGA4
LBP
IFNB1

KRAS
IL3
SNAI1
LOC106627981
SELE
PLAT
PTGS2
IL22
GSTM1
IGFBP5
CYBA
SOD1
CD40
CD27
ITGAL
TMEM67
S100A9
CD44
GAA
ATM
MIR142
KNG1
HFE
TNFSF13B
MIR130A
RET
MYPN
CSF2RB
CD14
MYL3
BPI
NPPA
CP
FIP1L1
NOS2
SOD2
TGIF1
RBP4
PTCH1
FGF10
PLA2G6
MIR34C
SREBF1
CD69
MYL2
MIR409
TP63
MIR338
MIR708
CASP8
MS4A1
SERPINB1
SLC11A1
MB
VWF
BMP2
ESR1
RAC2

SOD3
VDR
TNFSF11
SERPINC1
TH
AXL
ERF
IL4R
PAX2
CD81
ADRB3
GZMB
CASP1
FLNC-AS1
SELP
HDAC9
LYZ
EDN3
DHFR
MALT1
TNFSF12
EXOSC10
DNAH11
EPO
IFNA2
MIR15A
PRDM16
KITLG
HRH2
CLCN5
TNFRSF1B
CD1C
PLG
TIMP3
HAVCR1
PKHD1
LTF
CD1A
GLA
ALMS1
MIR146A
JAK3
CREBBP
NPC1
PRKAG2
APP
ABCB1
ASCL1
TACR1
IL1RAPL2
MTHFR
MYC
GSTP1
CYP2C19
TTR
HYDIN
CSF3R
CFI

IL12A
STAT1
RAC1
CCR3
CHIT1
GSTT1
MAPK14
LAMA3
LCN2
MAPK1
SCARB2
ACTA1
TRPV4
APOH
VIP
GJB2
CD80
CD86
EP300
FAM111B
CRYAB
KCNQ1
ADIPOQ
NAGLU
BRAF
ANXA5
PTGS1
GJA1
CPT2
SDC1
ITGB3
RARA
PADI4
PLAU
GATA6
IGF2
IRF5
NTRK1
MTOR
CD28
FCGR3A
SLC17A5
RSPH9
IFNGR1
MT-CYB
NTF3
TIMP2
ANO1
IL7
CRHR1
ITGB1
HLA-C
ERBB2
BAX
IL11
PECAM1
SST
NCAM1

BCL2
MIR25
COL2A1
DCN
EPRS1
DYNC2H1
CAT
TLR6
DNAH8
CSF2RA
NR3C1
NFE2L2
COL4A1
IL13RA1
MIR34A
MECP2
CC2D2A
IKBKG
HSPG2
CSF1
SRC
PLAUR
COL6A2
DNAH1
CDH2
DNAAF1
DNAAF11
NOTCH1
ANGPT2
RSPH1
MYH2
TYK2
CYP19A1
MDM2
EDNRA
GHRL
NAMPT
CTSB
TNFRSF8
RASGRP1
ADAM17
ANGPT1
MAPK8
CXCL13
MARCO
CYCS
CTSG
MIR181A2
PINK1
CFAP298
ENO2
APOA1
CDKN2A
ILK
CDKN1A
PRODH
KIF20A
PPARA

ABL1
HSPA4
CD55
C5
LEPR
SYP
CCR1
FCGR3B
RHOA
DPP4
IL17F
SYK
NCF4
TLR1
TLR7
HMGCR
KDR
TBX1
IDO1
CYP2D6
ACADVL
TRB
C4A
NPY
APOB
CST3
SETBP1
CCL21
LAMP1
IRF3
JAK1
HSP90AA1
KL
AARS1
TCIRG1
PGR
RAD51
FOXJ1
ENO1
GUSB
KRT7
NTRK3
AQP5
CCL19
EPHX1
PTX3
IKZF1
AP3D1
BSG
MCL1
XIAP
CREB1
JUP
G6PD
MMP13
CLU
GLI3
GDF15

SERPINF2
SETD2
HAVCR2
TARS1
PSMB8
CCL24
S100B
GFAP
CD163
TERF2
MME
PPBP
ITGAV
XDH
ABCA1
PARK7
ERCC6
COL4A2
CDH5
NLRC4
LIF
CTSD
IRF1
CHGA
NQO1
GBE1
VDAC1
TBX5
IGF2R
TARDBP
RAB8A
IGF1R
ACHE
MSN
SLC34A2
CCR8
MMP14
NOD1
H2AX
CXCL11
BMP7
TLL1
ANXA6
SIRT1
COL7A1
HSPA5
STX1A
MIR145
MIR223
CCL26
BRCA1
RELA
MICA
IL1RL1
IL12RB1
CTSL
FCN2
TNFAIP3

DNAJB13
MIR27A
CCL20
FGA
FABP3
RNPC3
HDAC4
PLA2G2A
HDAC2
CD209
NFKBIA
RUNX2
NPM1
EPX
PIK3CG
PLP1
TYMP
NAT2
F9
IGES
ITGA5
BRD4
CADM1
RPGRIP1L
TFRC
ATP13A2
HOTAIR
HPS6
LGALS9
HPS3
CYP2C9
F13A1
FLT4
HSPB1
PRG2
OSM
DEFB1
PPIG
OMP
LIPA
TXN
HRH1
CXCR6
DEFB103B
TUBB
MIR146B
TGFA
RAP1A
ITGA1
AKR1B1
ABCB7
HDAC8
PRKCA
KCNJ11
HBG2
PLA2G1B
C5AR1
PDPN

PARP1
PDE4A
CFLAR
AK2
CDC42
PRSS2
SPI1
EGR1
TNFSF14
F2RL1
VCAN
GAS5
ALPP
MECOM
FCN3
LAMP3
CLDN5
CCL7
NR1H2
ADK
RPS27A
FBN2
FURIN
SOX9
ADAMTSL1
HMOX2
IKBKB
IL12RB2
RPS6KB1
CTSH
SRXN1
IGFBP2
NOTCH3
ETS1
MKI67
PLA2G4A
NOX1
CGB3
B3GAT1
CTSK
GLRX
HABP2
GGT1
ITLN1
AGRN
GRN
ANXA2
IL27
CPS1
STC1
TNFRSF9
NRP1
IL16
FGB
RDX
LGALS1
DNASE1
COL11A1

TNFRSF4
F10
MET
FABP4
PRDX1
P2RY2
TUG1
MUTYH
PTGES
BPIFB1
FLI1
PLCG1
SLC4A1
GPRC5A
KLK3
CEBPB
LEPROT
CPQ
SP1
KIF1B
MASP2
PLOD1
C1QA
C1QC
C1QB
NR0B2
PTCH2
RAD54L
NFIA
DRC1
NPHP1
SEC61A1
NPHP3
CCDC39
RNF168
WDR19
