# Supplementary material for: Network Pharmacology Prediction and Molecular Docking-Based Strategy to Discover the Potential Pharmacological Mechanism of Wen-Yu-Jin against Pulmonary Fibrosis in a Mouse Model
Source: Evid Based Complement Alternat Med. 2022 Feb 10;2022:7753508. doi: 10.1155/2022/7753508 (PMC8853792; doi:10.1155/2022/7753508)
Supplement: Supplementary Materials — Supplementary Table S1: information about WYJ-related targets. Supplementary Table S2: information about PF-related targets. Supplementary Table S3: targets information at the intersection of drug targets and disease targets. Supplementary Table S4: 48 key targets of WYJ against PF. Supplementary Table S5: core gene information filtered according to the “betweenness,” “closeness,” and “degree” values. Supplementary-Molecular Docking: information about molecular docking. [file 7753508.f1.zip › 7753508.f1/Supplementary Table S3.pdf]

Supplementary Table S3 124 potential targets for WJY treatment on IPF

ABCB1
ABL1
ACE
ADAM17
ADRB2
ADRB3
AGTR1
AKR1B1
AKT1
ALOX5
APP
AR
ATP12A
ATP4A
BRAF
BRD4
C5AR1
CASR
CCND1
CCR4
CCR5
CD81
CMA1
CREBBP
CRHR1
CTSB
CTSD
CTSG
CTSH
CTSK
CTSL
CYP19A1
CYP2C9
DHFR
DPP4
EDNRA
EGFR
EP300
EPHX1
ESR1
F2
F3
FABP3
FABP4
FGFR1
G6PD
GSTP1
HDAC8
HMGCR
HMOX1
HRH1
HRH2
HSP90AA1
ICAM1
IDO1
IGF1R

IKBKB
IKBKG
IL1B
IL6
ITGAL
ITGB2
JAK1
JAK3
KCNJ11
KDR
LIPA
MAPK1
MAPK14
MAPK8
MCL1
MDM2
MET
MIF
MME
MMP1
MMP13
MMP2
MMP3
MMP8
MMP9
MPO
MTOR
MYLK
NAMPT
NFE2L2
NLRP3
NOS1
NOS2
NOS3
NR3C1
PARP1
PDGFRB
PGR
PLA2G1B
PLA2G2A
PLA2G6
PLCG1
PPARA
PPARG
PRKCA
PRKCD
PTGES
PTGS1
PTGS2
PTPN11
PTPRC
RASGRP1
RELA
REN
RET
RPS6KB1
SERPINE1
SIRT1

SRC
STAT3
STAT6
SYK
TACR1
TERT
TGFBR1
TLR4
TLR9
TYK2
