# Supplementary material for: Network Pharmacology Prediction and Molecular Docking-Based Strategy to Discover the Potential Pharmacological Mechanism of Wen-Yu-Jin against Pulmonary Fibrosis in a Mouse Model
Source: Evid Based Complement Alternat Med. 2022 Feb 10;2022:7753508. doi: 10.1155/2022/7753508 (PMC8853792; doi:10.1155/2022/7753508)
Supplement: Supplementary Materials — Supplementary Table S1: information about WYJ-related targets. Supplementary Table S2: information about PF-related targets. Supplementary Table S3: targets information at the intersection of drug targets and disease targets. Supplementary Table S4: 48 key targets of WYJ against PF. Supplementary Table S5: core gene information filtered according to the “betweenness,” “closeness,” and “degree” values. Supplementary-Molecular Docking: information about molecular docking. [file 7753508.f1.zip › 7753508.f1/Supplementary Table S4.pdf]

Supplementary Table S4 48 key targets of WYJ against IPF

Target name	Betweenness Centra	Closeness Centra	Degree
IL6	0.10215744	0.83108108	98
AKT1	0.07180425	0.78343949	89
EGFR	0.04777266	0.73214286	79
MAPK1	0.03989939	0.71929825	76
SRC	0.02898867	0.69886364	71
STAT3	0.02623321	0.69101124	70
PTGS2	0.03474508	0.69491525	70
MAPK8	0.02093088	0.68715084	69
IL1B	0.03114078	0.68715084	68
MMP9	0.03146996	0.66486486	64
TLR4	0.01993166	0.65079365	59
HSP90AA1	0.01920591	0.6373057	56
MMP2	0.01241093	0.63076923	54
CCND1	0.0114142	0.63076923	53
MAPK14	0.00750973	0.63076923	53
MTOR	0.0109869	0.62755102	53
NOS3	0.01997073	0.63402062	52
PPARG	0.0343203	0.63076923	51
ICAM1	0.01247068	0.62755102	51
ESR1	0.00739533	0.615	49
SIRT1	0.01497138	0.615	47
RELA	0.0052015	0.60294118	45
PTPRC	0.00881161	0.6	44
MPO	0.015004	0.59708738	42
KDR	0.00423423	0.58571429	41
ACE	0.01306016	0.59708738	41
EP300	0.00932664	0.59134615	40
SERPINE1	0.00577073	0.58851675	40
APP	0.00888037	0.58293839	37
HMOX1	0.00949351	0.57746479	36
REN	0.0088579	0.58293839	36
STAT6	0.00237573	0.57209302	35
MDM2	0.00312134	0.57209302	34
AR	0.00273555	0.57209302	34
TLR9	0.0026224	0.56422018	33
JAK1	0.00216645	0.55656109	32
MMP1	0.00270628	0.56682028	32
MMP3	0.00226773	0.55909091	31
F2	0.00773739	0.56682028	31
NOS2	0.00305134	0.56422018	30
CREBBP	0.00350145	0.55656109	30
AGTR1	0.00417234	0.56164384	29
PRKCA	0.00628084	0.55405405	28
NR3C1	0.00782177	0.55405405	27
PRKCD	0.00372331	0.53947368	25
SYK	0.00282708	0.5371179	25
ALOX5	0.00350515	0.54424779	24
CCR5	0.00367484	0.5371179	23
