# Supplementary material for: Network Pharmacology Prediction and Molecular Docking-Based Strategy to Discover the Potential Pharmacological Mechanism of Wen-Yu-Jin against Pulmonary Fibrosis in a Mouse Model
Source: Evid Based Complement Alternat Med. 2022 Feb 10;2022:7753508. doi: 10.1155/2022/7753508 (PMC8853792; doi:10.1155/2022/7753508)
Supplement: Supplementary Materials — Supplementary Table S1: information about WYJ-related targets. Supplementary Table S2: information about PF-related targets. Supplementary Table S3: targets information at the intersection of drug targets and disease targets. Supplementary Table S4: 48 key targets of WYJ against PF. Supplementary Table S5: core gene information filtered according to the “betweenness,” “closeness,” and “degree” values. Supplementary-Molecular Docking: information about molecular docking. [file 7753508.f1.zip › 7753508.f1/Supplementary Table S5.pdf]

Supplementary Table S5

The topological feature values of the key targets in Compound-Target-Pathway network

Target nar	Betweenn	Closeness	Degree
STAT3	0.024531	0.972222	34
SRC	0.022467	0.972222	34
IL6	0.025059	0.972222	34
MAPK1	0.018606	0.945946	33
AKT1	0.015892	0.921053	32
EGFR	0.014583	0.897436	31
MAPK8	0.012962	0.897436	31
MAPK14	0.011751	0.875	30
IL1B	0.014353	0.853659	29
MTOR	0.01329	0.853659	29
PTGS2	0.006919	0.853659	29
MMP9	0.010099	0.853659	29
TLR4	0.012624	0.853659	29
RELA	0.013874	0.853659	29
HSP90AA1	0.01137	0.833333	28
CCND1	0.007317	0.813953	27
SIRT1	0.005228	0.795455	26
MMP2	0.003474	0.795455	26
ICAM1	0.009665	0.795455	26
NOS3	0.005593	0.777778	25
ESR1	0.003951	0.777778	25
STAT6	0.006382	0.76087	24
EP300	0.005668	0.744681	23
TLR9	0.005974	0.744681	23
KDR	0.003056	0.729167	22
JAK1	0.010756	0.714286	21
HMOX1	0.001264	0.7	20
SERPINE1	0.001051	0.7	20
MMP3	0.0036	0.7	20
MDM2	0.001345	0.686275	19
NOS2	0.000702	0.673077	18
CREBBP	0.001858	0.660377	17
PRKCD	0.001486	0.614035	13
SYK	0.001672	0.614035	13
PRKCA	0.002195	0.603448	12
CCR5	0.000309	0.57377	9
